# Sex- and age-specific associations between body composition markers and estimated glomerular filtration rate–results of a population-based study

**DOI:** 10.1093/ckj/sfag261

**Published:** 2026-08-07

**Authors:** Marlene Agnes Günther, Till Ittermann, Henry Völzke, Sylvia Stracke, Karlhans Endlich, Robin Bülow, Matthias Nauck, Mats Wiese, Ali Aghdassi, Marcello Ricardo Paulista Markus, Sabrina von Rheinbaben

**Affiliations:** Department of Internal Medicine A, University Medicine, Greifswald, Germany; Institute for Community Medicine, University Medicine, Greifswald, Germany; Institute for Community Medicine, University Medicine, Greifswald, Germany; Department of Internal Medicine A, University Medicine, Greifswald, Germany; Institute of Anatomy and Cell Medicine, University Medicine, Greifswald, Germany; Institute for Diagnostic Radiology and Neuroradiology, University Medicine, Greifswald, Germany; Institute of Clinical Chemistry and Laboratory Medicine, University Medicine, Greifswald, Germany; German Centre for Cardiovascular Research (DZHK), partner site Greifswald, Greifswald, Germany; Department of Food, Nutrition, Facilities, University of Applied Sciences Münster, Germany; Department of Internal Medicine A, University Medicine, Greifswald, Germany; Department of Internal Medicine B, University Medicine Greifswald, Greifswald, Germany; German Centre for Cardiovascular Research (DZHK), partner site Greifswald, Greifswald, Germany; Department of Internal Medicine C, University Medicine Greifswald, Germany

**Keywords:** age, body composition, chronic kidney disease, kidney function, sex difference

## Abstract

**Background:**

Recent studies have indicated that body composition may affect kidney function. The aim of this study was to examine the sex- and age-related association between body composition markers and the estimated glomerular filtration rate (eGFR).

**Methods:**

Analyses were based on data from the population-based ‘Study of Health in Pomerania’ (SHIP), including data from 4211 individuals enrolled in the SHIP Trend-0 cohort. Body composition markers included measurements from classic anthropometry (body mass index, waist-to-hip ratio), body impedance analysis and magnetic resonance imaging (subcutaneous, hepatic and visceral fat mass). The eGFR was estimated using creatinine- and Cystatin C-based formulas. Cross-sectional analysis was performed using linear regression models adjusted for confounding variables.

**Results:**

Generally, higher values of body composition markers were associated with lower eGFR levels for all formulas, while higher relative fat-free mass was associated with higher eGFR. The effects were more pronounced in women compared to men. Regarding age dependency, increased values of body or fat mass were associated with higher eGFR in younger age groups, but with lower eGFR in older age groups. In contrast, higher relative fat-free mass was associated with increased eGFR in older age groups.

**Conclusions:**

Increased body and fat mass, regardless of distribution, and a lower percentage of muscle mass are associated with lower eGFR especially in women and older individuals.

## Graphical Abstract

**Graphical Abstract:**
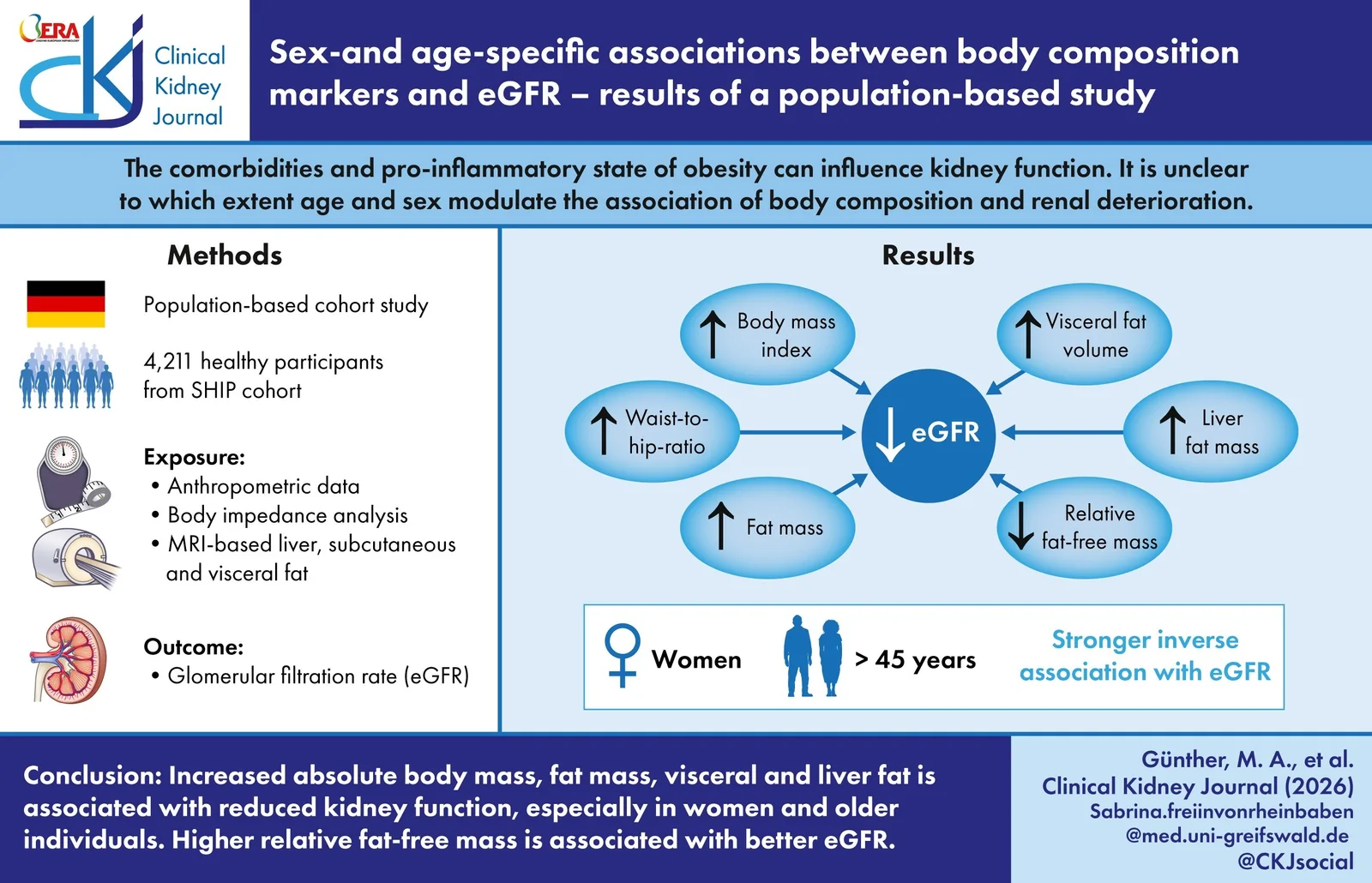

KEY LEARNING POINTS **What was known:**

- Obesity and its comorbidities, such as diabetes or hypertension, are important risk factors for a decline in kidney function.
- Elevated visceral or liver fat is associated with lower eGFR in individuals with diabetes or obesity.

**This study adds:**

- Higher values of various body composition markers are associated with lower eGFR.
- The association between body composition markers and eGFR appears to be more pronounced in women and older individuals.
- Higher relative fat-free mass is associated with better kidney function.

**Potential impact:**

- As women and older individuals in our study were most vulnerable to an increase in body composition markers, this underlines the importance of sex- and age-specific research.

## INTRODUCTION

In north-east Germany, more than 17% of the population is affected by chronic kidney disease, 22.4% are considered obese [1, 2]. Obesity and its comorbidities, such as type 2 diabetes or arterial hypertension, are relevant risk factors for developing kidney damage. However, the pathophysiology behind obesity-related deterioration in renal function has not yet been conclusively clarified. Some studies indicate that the pro-inflammatory effect of adipose tissue could result in kidney damage, others stress the importance of insulin resistance [3–7]. Elevated visceral and liver fat exacerbate the process, while muscle mass tends to have a beneficial effect [8–10]. However, glomerular hyperfiltration is a well-known adverse effect of obesity [11]. But even in non-obese individuals, an increased body mass index (BMI) was associated with higher glomerular pressure [12]. Accordingly, an elevated BMI was associated with reduced estimated glomerular filtration rate (eGFR) in a longitudinal, population-based German study [13]. Apart from the body composition itself, sex and the ageing process appear to play a role. For example, men tend to have more metabolically active visceral fat, whereas premenopausal women tend to have more metabolically inactive subcutaneous fat. After menopause, fat tissue is redistributed, resembling more closely the male distribution pattern and unfavourable metabolic profile [14, 8]. As a result, some authors found an association between higher BMI and lower eGFR in both sexes, others found obesity to be associated with decreased eGFR only in women [13, 15]. Therefore, the aim of this study was to investigate the association between body composition and eGFR with respect to sex and age in a healthy population.

## MATERIALS AND METHODS

### Study population

Our analysis used data from the population-based cohort study ‘Study of Health in Pomerania (SHIP)’, conducted by the University Medicine Greifswald in North-East Germany [16]. The study protocol received approval from the Ethics Committee at the University of Greifswald and adheres to the Declaration of Helsinki (approval number: III UV 73/01). Participants aged 20–79 years were randomly selected from population registries [17]. In the SHIP-TREND-0-cohort, 4420 individuals participated from 2008 until 2012 after providing written consent. After excluding pregnant participants and individuals with missing exposure or outcome data, 4211 participants remained for analysis. Analyses regarding the magnetic resonance imaging (MRI)-based markers visceral/subcutaneous and liver fat were analysed in subpopulations (*n* = 1941 and *n* = 1890). All examinations were carried out at the University of Greifswald by specially trained experts in a standardized manner. The data collection was based on computer-assisted interviews or questionnaires completed by the participants themselves. Laboratory analyses were performed at the Institute of Laboratory Medicine of the University Medicine Greifswald under internal and external quality assurance meeting the quality requirements of the German Medical Association on Quality Assurance in Medical Laboratory Examinations [18].

### Anthropometric data

Using calibrated measuring devices, height was measured to the nearest 1 cm, the waist and hip circumference (HC) to the nearest 0.5 cm and the body weight to the nearest 0.1 kg [19]. The measurement of waist circumference (WC) was taken at the midway between the lower rib margin and the iliac crest. Furthermore, the HC was assessed either on the buttocks or horizontally at the point of largest lateral dimension [19]. To calculate the waist-to-hip ratio (WHR), WC was divided by HC. BMI was calculated as BMI = weight in kg/(height in m)², body surface area (BSA) as BSA = 0.007184 × Height^0.725^ × Weight^0.425^ (DuBois).

### Body impedance analysis

A multifrequency Nutriguard-M device (Data Input GmbH, Pöcking, Germany) and NutriPlus software (version 5.4.1, Data Input GmbH, Pöcking, Germany) were used to carry out body impedance analysis (BIA). The measuring electrodes were attached to the dorsal side of the dominant hand, wrist, ankle, and foot while participants were in the supine position. Following the manufacturer’s specifications, resistance (R) and reactance (Xc) were measured using electrical currents of 800 µA at 5, 50, and 100 kHz [20]. Relative fat-free mass (FFM%) was given as a percentage, fat mass index (FMI) was calculated as FMI = fat mass/height², fat-free mass index (FFMI) as FFMI = FEM/height² and body cell mass index (BCMI) as BCMI = body cell mass/height².

### MRI data

MRI Data were acquired following a standardized MRI protocol [21]. A 1.5-T MR imager (Magnetom Avanto, Siemens Medical Systems, Erlangen, Germany) was used for native whole-body MRI. Five phased array surface coils were attached to the head, collar, abdomen, pelvis and lower extremities. For MRI of the abdomen, we used ax T2 FS (BLADE) sequence, with a voxel size of 1.6 × 1.6 × 6.0 and a scan time of 1:16 min. For whole-body MRI, the coronal TRIM sequence (five stations) was used for sequence, with a voxel size of 1.6 × 1.6 × 5.0 and a scan time of 12:09 min [21]. The quantification of subcutaneous fat tissue (SFT) and visceral fat tissue (VFT) was carried out by measuring the region of interest (ROI) between the left diaphragm and bladder using a plug-in from the existing SHIP-Trend-cohort MRI sequences [22]. We determined the liver fat content (LFC) using a coded three-echo MRI of the liver. The acquired MRI data were subsequently post-processed and the proton density of the fat fractions, measured by ROI in the centre of the liver, was registered [23].

### Glomerular filtration

Serum creatinine and serum Cystatin C concentrations were measured by enzymatic and nephelometric methods (Dimension VISTA, Siemens Healthcare Diagnostics, Eschborn, Germany). The glomerular filtration rate was estimated using the Chronic Kidney Disease Epidemiology Collaboration (CKD-EPI) 2009 formula for creatinine (CKD-EPI_crea_) [24] or 2012 formula for creatinine/Cystatin C (CKD-EPI_crea+cys_) [25] and the European Kidney Function Consortium (EKFC) 2021 equation for creatinine (EKFC_crea_) [26] or the 2023 formula for Cystatin C (EKFC_cys_) [27] as well as the 2026 equation for creatinine/Cystatin C (EKFC_crea+cys_) [28] (Supplementary Materials).

### Statistics

Characteristics of the study population were reported stratified by sex-specific quartiles of WC as median with interquartile range (continuous data) or as absolute numbers and percentages (categorical data). Exposure variables were standardized per standard deviation. Associations between body composition markers with eGFR were analysed by linear regression models adjusted for age, sex, smoking status, educational level, type 2 diabetes, fasting glucose, physical activity, dietary intake, C-reactive protein, and alcohol consumption. Afterwards, we repeated the multivariable analyses stratified by sex. A *P* value <.05 was considered statistically significant. Interactions with age were considered by introducing interaction terms of age with the respective exposure variable. If the interaction was significant (*P* < .1), age-specific effects of body composition markers on eGFR were visualized based on the corresponding interaction model. Our analyses were conducted in the statistical software STATA 19.5 (College Station, TX; USA).

## RESULTS

### Multivariable analyses

A full overview of all the results can be found in the Supplementary Materials. Characteristics of the study population are shown in Table 1. We observed significant associations between body composition markers and eGFR for all equations (Table 2). Generally, higher values of BMI, BSA, and WC were associated with lower eGFR. Regarding BIA, we found that individuals with an increased mass had a decreased eGFR, regardless of the body compartment. In contrast, higher FFM% was associated with increased eGFR. Additionally, a higher volume of VFT and LFC was associated with a lower eGFR across almost all equations. For SFT, this effect was shown only for Cystatin C-based and combined formulas.

**Table 1:** Characteristics of the study population stratified by sex-specific quartiles of waist circumference.

	Waist circumference				
	Q1 [74 cm (70; 84)]	Q2 [83 cm (79; 92)]	Q3 [94 cm (88; 101)]	Q4 [108 cm (102; 114)]	P*
*N*	1099 (25.3%)	1079 (24.9%)	1090 (25.1%)	1069 (24.6%)	
Age in years	39 (30; 49)	51 (40; 61)	58 (46; 68)	60 (51; 69)	<.001
Sex					.962
Male	535 (48.7%)	519 (48.1%)	525 (48.2%)	525 (49.1%)	
Female	564 (51.3%)	560 (51.9%)	565 (51.8%)	544 (50.9%)	
Body surface area; m²	1.77 (1.64; 1.93)	1.86 (1.74; 2.00)	1.95 (1.81; 2.08)	2.07 (1.93; 2.22)	<.001
Body mass index; kg/m²	22.8 (21.2; 24.7)	26.0 (24.5; 27.5)	29.1 (27.6; 30.7)	33.6 (31.4; 36.8)	<.001
Waist to hip ratio; %	82 (77; 87)	87 (81; 94)	91 (84; 97)	95 (88; 102)	<.001
Fat mass index; kg/m²	5.2 (4.3; 6.2)	7.0 (5.9; 8.3)	8.8 (7.5; 10.6)	11.9 (9.7; 14.6)	<.001
Fat-free mass index; kg/m²	17.4 (15.9; 19.2)	18.5 (16.9; 20.7)	19.9 (18.1; 22.1)	21.7 (19.8; 23.9)	<.001
Body cell mass index; kg/m²	9.1 (8.1; 10.7)	9.7 (8.7; 11.0)	10.2 (9.1; 11.7)	11.1 (9.9; 12.6)	<.001
Relative fat-free mass; %	77 (73; 81)	72 (68; 77)	69 (63; 74)	64 (58; 71)	<.001
Subcutaneous fat; l	4.57 (3.74; 5.72)	6.91 (5.75; 7.99)	8.96 (7.48; 10.7)	12.0 (9.72; 14.7)	<.001
Visceral fat; l	1.43 (0.82; 2.52)	3.04 (1.94; 4.58)	4.79 (3.37; 6.54)	6.66 (5.02; 8.59)	<.001
Liver fat; %	2.3 (1.9; 3.1)	3.4 (2.3; 5.6)	5.3 (3.3; 10.1)	9.7 (5.8; 17.4)	<.001
Creatinine; µmol/l	74 (65; 85)	75 (65; 86)	78 (67; 90)	80 (70; 93)	<.001
Cystatin C; mg/l	0.66 (0.60; 0.74)	0.69 (0.62; 0.76)	0.73 (0.66; 0.83)	0.78 (0.69; 0.90)	<.001
eGFR (EKFC_crea+cys_); ml/min/1.73 m²	107 (96; 115)	96 (84; 108)	89 (76; 100)	84 (73; 96)	<.001
eGFR (CKD-Epi_crea+cys_); ml/min/1.73 m²	112 (101; 121)	105 (93; 116)	97 (84; 109)	93 (78; 105)	<.001

Data are expressed as median, 25th, and 75th percentile (continuous data) or as absolute numbers and percentages (categorical data). Exposure variables were standardised per standard deviation.

Q, quartile of waist circumference with median and 25th, and 75th percentile; BMI, body mass index; eGFR, estimated glomerular filtration rate; EKFC, European Kidney Function Consortia equation; CKD-EPI, Chronic Kidney Disease Epidemiology Collaboration equation; crea + cys, Cystatin C-and creatinine-based formula.

*P-values derived from Kruskal-Walllis-tests (continuous data) or χ2-tests (categorical data).

**Table 2:** Associations of body composition markers with estimated glomerular filtration rate (eGFR).

	EKFC_crea+cys_ β (95%-CI)	CKD-Epi_crea+cys_ β (95%-CI)
Body surface area; m^2^	−1.25 (−1.59; −0.91)*	−2.11 (−2.64; −1.59)*
Body mass index; kg/m^2^	−1.00 (−1.29; −0.70)*	−1.65 (−2.10; −1.19)*
Waist circumference; cm	−1.04 (−1.38; −0.70)*	−1.82 (−2.35; −1.30)*
Waist-to-hip ratio	−0.31 (−0.71; 0.10)	−0.58 (−1.20; 0.04)
Fat mass index; kg/m^2^	−1.01 (−1.33; −0.70)*	−1.76 (−2.24; −1.27)*
Fat-free mass index; kg/m^2^	−1.13 (−1.51; −0.76)*	−1.75 (−2.33; −1.17)*
Body cell mass index; kg/m^2^	−0.91 (−1.27; −0.54)*	−1.20 (−1.76; −0.64)*
Relative fat-free mass; %	0.78 (0.41; 1.15)*	1.47 (0.89; 2.04)*
Subcutaneous fat; l	−0.61 (−1.02; −0.21)*	−1.28 (−1.91; −0.64)*
Visceral fat; l	−0.86 (−1.36; −0.36)*	−1.51 (−2.29; −0.73)*
Liver fat; %	−0.85 (−1.24; −0.46)*	−1.28 (−1.90; −0.67)*

Results are reported as standardised beta coefficients derived from linear regression models adjusted for age, sex, smoking status, years of education, diabetes, fasting glucose, alcohol, physical activity, dietary intake and C-reactive protein. Exposure variables were standardised per standard deviation.

EKFC, European Kidney Function Consortia equation; CKD-EPI, Chronic Kidney Disease Epidemiology Collaboration equation; crea + cys, Cystatin C-and creatinine-based formula; CI, confidence interval.

*P < .05.

### Sex-specific associations

Effect sizes were stronger in women compared to those in men and were more pronounced for CKD-EPI than for EKFC equations (Tables 3 and 4). In women, an increased body or fat mass, independent of measurement method, was associated with a lower eGFR across almost every equation. Additionally, an increased FFM% showed a higher eGFR. With regard to men, these results were inconsistent.

**Table 3:** Characteristics of the study population stratified by sex.

	Male	Female	P*
*N*	2104 (48.5%)	2233 (51.5%)	
Age in years	53 (40; 65)	52 (39; 63)	.011
Body surface area; m²	2.04 (1.93; 2.16)	1.78 (1.68; 1.90)	<.001
Body mass index; kg/m²	28.2 (25.5; 31.2)	26.7 (23.4; 30.9)	<.001
Waist-to-hip ratio; %	95 (90; 100)	82 (78; 87)	<.001
Fat mass index; kg/m²	6.7 (5.3; 8.4)	9.1 (6.8; 12.0)	<.001
Fat-free mass index; kg/m²	21.4 (19.8; 23.0)	17.6 (16.4; 19.0)	<.001
Body cell mass index; kg/m²	11.5 (10.5; 12.6)	8.9 (8.2; 9.8)	<.001
Relative fat-free mass; %	76 (72; 80)	66 (61; 71)	<.001
Subcutaneous fat; l	6.51 (4.91; 8.53)	8.31 (6.19; 11.13)	<.001
Visceral fat; l	5.16 (3.12; 7.13)	2.49 (1.27; 3.95)	<.001
Liver fat; %	4.8 (2.8; 9.1)	3.2 (2.1; 6.8)	<.001
Creatinine; µmol/l	85 (76; 95)	69 (61; 77)	<.001
Cystatin C; mg/l	0.73 (0.66; 0.82)	0.69 (0.62; 0.78)	<.001
eGFR (EKFC_crea+cys_); ml/min/1.73 m²	94 (80; 106)	95 (81; 107)	.078
eGFR (CKD-Epi_crea+cys_); ml/min/1.73 m²	104 (90; 115)	101 (87; 113)	.002

Data are expressed as median, 25th, and 75th percentile (continuous data) or as absolute numbers and percentages (categorical data). Exposure variables were standardised per standard deviation.

ACR, urinary albumin creatinine ratio; BMI, body mass index; eGFR, estimated glomerular filtration rate; EKFC, European Kidney Function Consortia equation; CKD-EPI, Chronic Kidney Disease Epidemiology Collaboration equation; crea + cys, Cystatin C-and creatinine-based formula.

*P-values derived from Kruskal-Walllis-tests (continuous data) or χ2-tests (categorical data).

**Table 4:** Associations of body composition markers with estimated glomerular filtration rate (eGFR) in females and males.

	EKFC_crea+cys_ β (95%-CI)		CKD-Epi_crea+cys_ β (95%-CI)	
	Female	Male	Female	Male
Body surface area; m^2^	−1.62 (−2.11; −1.14)*	−0.89 (−1.37; −0.41)*	−2.79 (−3.53; −2.05)*	−1.48 (−2.23; −0.73)*
Body mass index; kg/m^2^	−1.24 (−1.62; −0.86)*	−0.61 (−1.08; −0.13)*	−2.07 (−2.66; −1.49)*	−0.99 (−1.73; −0.26)*
Waist-to-hip ratio	−0.70 (−1.28; −0.11)*	−0.01 (−0.57; 0.55)	−1.22 (−2.12; −0.33)*	−0.06 (−0.93; 0.80)
Fat mass index; kg/m²	−1.23 (−1.61; −0.85)*	−0.42 (−1.01; 0.16)	−2.07 (−2.66; −1.49)*	−0.92 (−1.83; −0.02)*
Fat-free mass index; kg/m²	−1.80 (−2.40; −1.20)*	−0.69 (−1.17; −0.20)*	−2.91 (−3.82; −1.99)*	−0.99 (−1.74; −0.24)*
Body cell mass index; kg/m²	−1.86 (−2.44; −1.27)*	−0.21 (−0.68; 0.26)	−2.85 (−3.74; −1.95)*	−0.04 (−0.77; 0.69)
Relative fat-free mass; %	1.13 (0.65; 1.61)*	0.14 (−0.46; 0.75)	1.98 (1.25; 2.71)*	0.54 (−0.39; 1.48)
Subcutaneous fat; l	−0.69 (−1.23; −0.15)*	−0.41 (−1.05; 0.23)	−1.36 (−2.18; −0.53)*	−1.07 (−2.09; −0.05)*
Visceral fat; l	−2.03 (−2.97; −1.09)*	−0.46 (−1.04; 0.11)	−3.43 (−4.88; −1.98)*	−0.83 (−1.74; 0.08)
Liver fat; %	−1.35 (−1.93; −0.77)*	−0.47 (−1.00; 0.06)	−2.10 (−3.01; −1.19)*	−0.65 (−1.50; 0.19)

Results are reported as standardised beta coefficients derived from linear regression models for age, smoking status, years of education, diabetes, fasting glucose, alcohol, physical activity, dietary intake and C-reactive protein. Exposure variables were standardised per standard deviation.

EKFC, European Kidney Function Consortia equation; CKD-EPI, Chronic Kidney Disease Epidemiology Collaboration equation; crea + cys, Cystatin C + creatinine; CI, confidence interval.

*P < .05.

### Effect modifications by age

**Figure 1:**
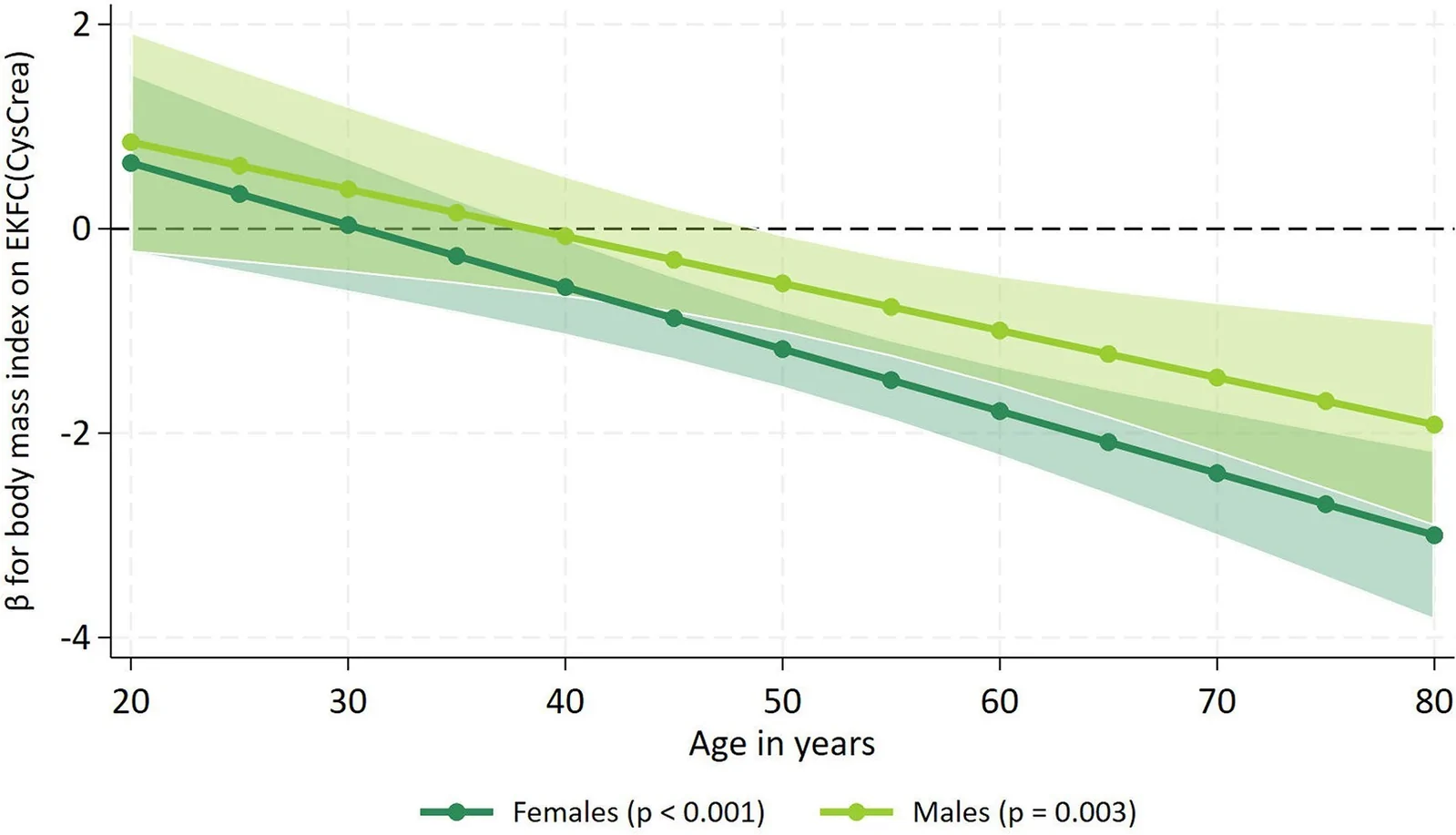
Standardized beta coefficients for BMI and eGFR (EKFC_crea+cys_) in different age groups for both sexes (females in dark green, males in light green). Results are reported as mean with confidence interval for each age group. Linear regression model adjusted for age, smoking status, years of education, diabetes, fasting glucose, alcohol, physical activity, dietary intake and C-reactive protein, exposure variables standardized per standard deviation. BMI, body mass index; eGFR, estimated glomerular filtration rate; EKFC (CysCrea), European Kidney Function Consortia equation using Cystatin C + creatinine.

**Figure 2:**
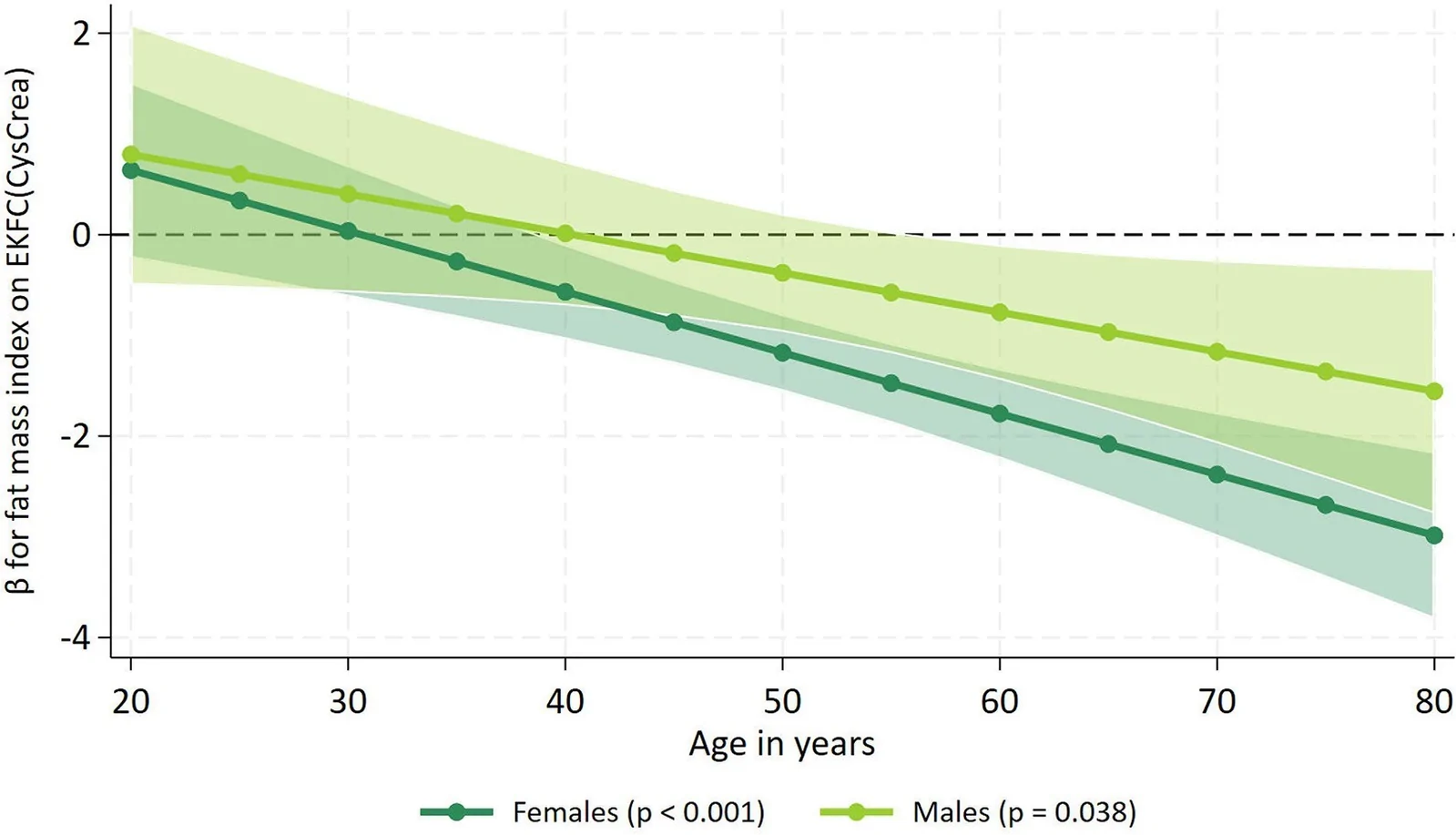
Standardized beta coefficients for fat mass index (FMI) and eGFR (EKFC_crea+cys_) in different age groups for both sexes (females in dark green, males in light green). Results are reported as mean with confidence interval for each age group. Linear regression model adjusted for age, smoking status, years of education, diabetes, fasting glucose, alcohol, physical activity, dietary intake and C-reactive protein, exposure variables standardized per standard deviation. eGFR, estimated glomerular filtration rate; EKFC (CysCrea), European Kidney Function Consortia equation using Cystatin C + creatinine.

**Figure 3:**
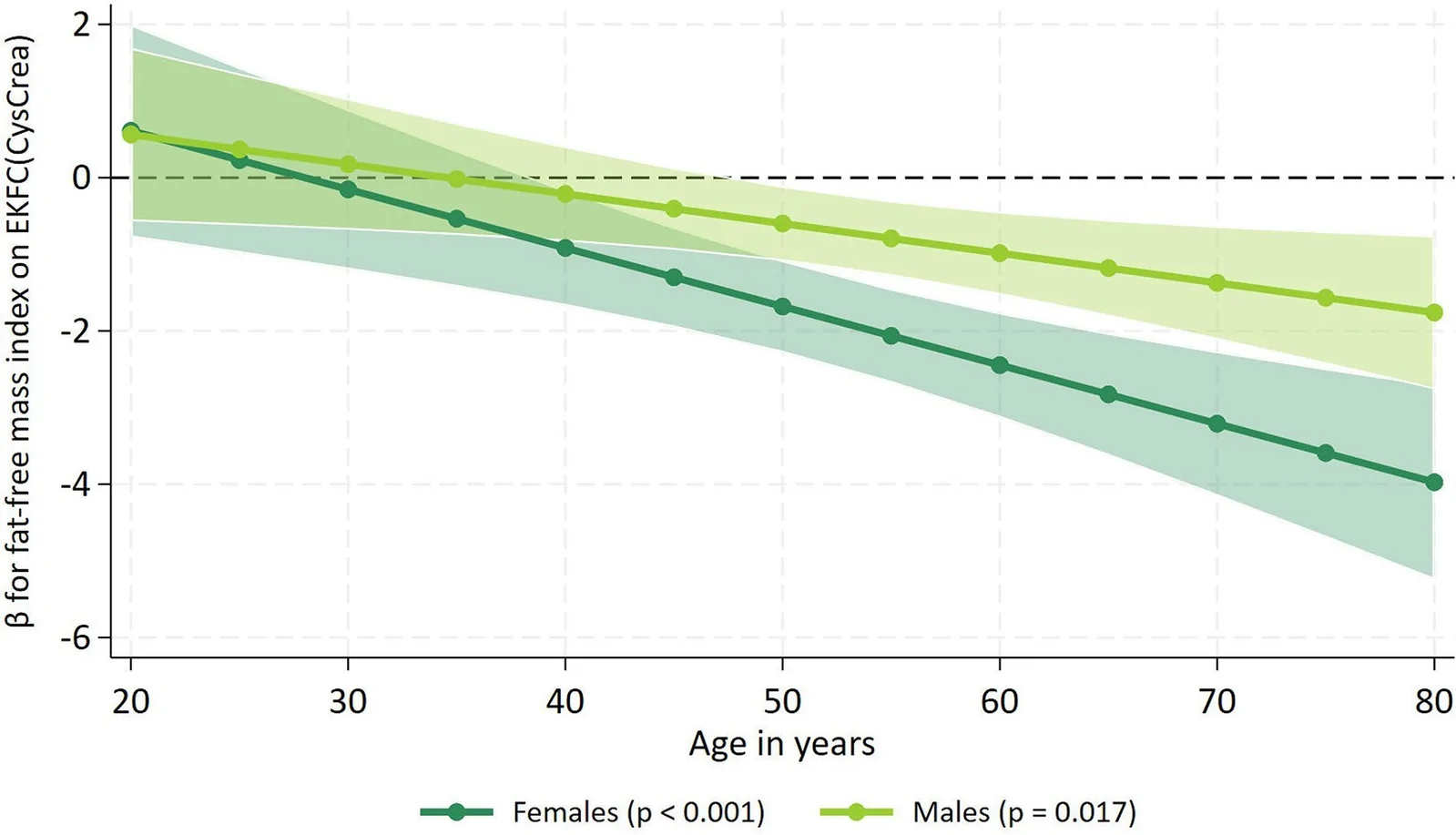
Standardized beta coefficients for fat-free mass index (FFMI) and eGFR (EKFC_crea+cys_) in different age groups for both sexes (females in dark green, males in light green). Results are reported as mean with confidence interval for each age group. Linear regression model adjusted for age, smoking status, years of education, diabetes, fasting glucose, alcohol, physical activity, dietary intake and C-reactive protein, exposure variables standardized per standard deviation. eGFR, estimated glomerular filtration rate; EKFC (CysCrea), European Kidney Function Consortia equation using Cystatin C + creatinine.

**Figure 4:**
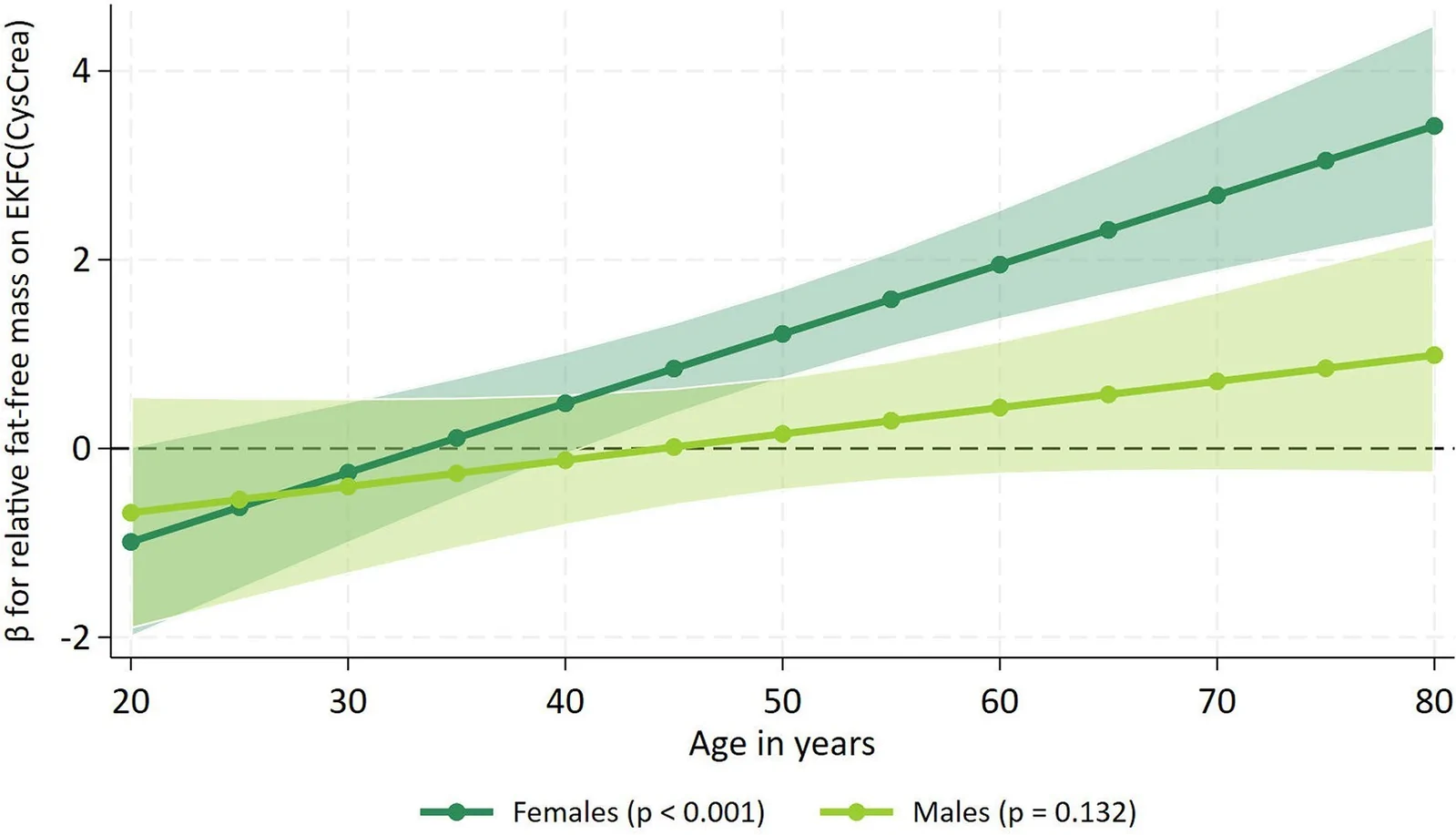
Standardized beta coefficients for relative fat-free mass (FFM%) and eGFR (EKFC_crea+cys_) in different age groups for both sexes (females in dark green, males in light green). Results are reported as mean with confidence interval for each age group. Linear regression model adjusted for age, smoking status, years of education, diabetes, fasting glucose, alcohol, physical activity, dietary intake and C-reactive protein, exposure variables standardized per standard deviation. eGFR, estimated glomerular filtration rate; EKFC (CysCrea), European Kidney Function Consortia equation using Cystatin C + creatinine.

**Figure 5:**
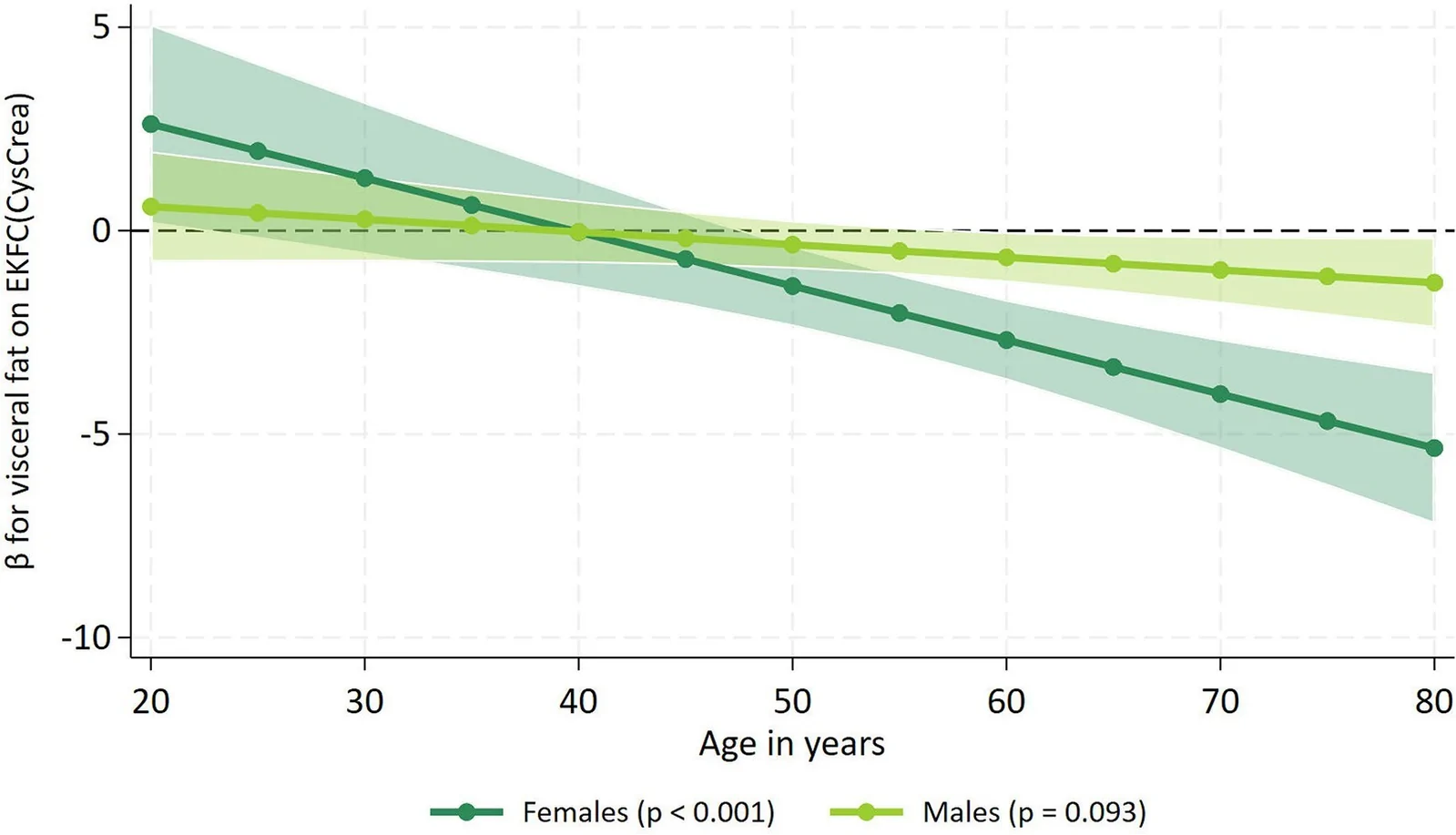
Standardized beta coefficients for visceral fat mass and eGFR (EKFC_crea+cys_) in different age groups for both sexes (females in dark green, males in light green). Results are reported as mean with confidence interval for each age group. Linear regression model adjusted for age, smoking status, years of education, diabetes, fasting glucose, alcohol, physical activity, dietary intake and C-reactive protein, exposure variables standardized per standard deviation. eGFR, estimated glomerular filtration rate; EKFC (CysCrea), European Kidney Function Consortia equation using Cystatin C + creatinine.

In general, women exhibited larger effect sizes than men (Figs 1–5). Overall, we observed positive beta coefficients for the association between anthropometric body composition markers and eGFR in younger age groups suggesting a higher eGFR in pre-obesity or even obesity. In age groups above 40 years of age, the beta coefficients of the anthropometric body composition markers became negative indicating that higher values of the body composition markers were associated with lower eGFR levels. The effect was similar for both women and men. Regarding BIA, FMI and FFMI had comparable results to the anthropometric body composition markers. An exception to this was BCMI, which showed an association between increased values and higher eGFR in men but not in women. However, a higher FFM% was associated with lower eGFR in younger age groups, but was associated with a higher one in groups older than 40 years for both sexes. SFT, VFT, and LFC showed similar results to anthropometric variables.

## DISCUSSION

In women, an increased body or fat mass was associated with a decreased eGFR, whereas higher FFM% was associated with higher eGFR. The results for men were inconsistent. Overall, with the exception of FFM%, higher values of body composition markers were associated with a higher eGFR in younger age groups but with a lower eGFR in older ones. In general, effect sizes were stronger in women.

Most studies investigating the association between obesity and kidney function rely on BMI, WC, or WHR to define overweight. As a result, the connection between higher anthropometric parameters and reduced eGFR is well established [3, 4, 29]. For example, Herold *et al*. demonstrated that an increased BMI was associated with a decreased eGFR [13]. Additionally, the association between higher fat mass and lower eGFR was also mentioned [30]. Recently, other characteristics of obesity, e.g. the distribution of fatty tissue measured via MRI, have been identified as important risk factors. Some authors described a larger VFT as a risk factor for diabetic kidney disease or reduced eGFR [8, 31]. Mueller-Peltzer *et al*. found this association even in a healthy population [32]. In particular, this finding has been attributed to increased pro-inflammatory leptin and its profibrotic effects on renal tissue as well as the release of adipokines, whose accumulation leads to insulin resistance [7, 9, 33]. In line with our results, other studies also saw an association between a high amount of LFC and a reduced eGFR/CKD, whether it was in the absence or presence of metabolic syndrome. The authors acknowledged similar processes to those observed with VFT emphasizing the pro-inflammatory state caused by the elevated LFC [10]. Nevertheless, other authors did not find an association between SFT and eGFR, and some even suggested a partially protective effect of subcutaneous fat in metabolically unhealthy obese [8, 30, 32].

In our study, women showed higher effect sizes in comparison to men. Waas *et al*. found a stronger association between an elevated BMI and a lower eGFR in women [15]. Leone *et al*. showed that an increased VFT was associated with reduced eGFR only in women, but showed greater effect sizes for women than men regarding fat mass and FEM [30]. In contrast to our study, Asakawa *et al*. found elevated VFT to be associated with diabetic kidney disease only in men [34]. Moh *et al*. demonstrated that an increase in BMI, weight and VFT in diabetic patients was associated with a decline in eGFR in men [35]. Both studies were conducted among older individuals with long-standing diabetes, and, thus, may not apply to our population-based study. Although our study lacks data on menopausal status, we believe that the beneficial premenopausal hormone profile may play a role in the sex-specific differences of our results. As the young median age of our female subjects may correspond to the early menopausal transition, many participants might not have fully, or not at all, experienced the drop in oestrogen and its accompanying redistribution from SFT to VFT. We hypothesize that the development of a higher proportion of e.g. VFT or fat mass even at a younger age, may be especially harmful, since its onset falls within a period still under the beneficial influence of higher oestrogen levels. Further studies are required to investigate this issue.

In our study, an increased FFM% was associated with a higher eGFR, especially in women. Leone et al. observed a similar effect, even after including the distribution of VFT and SFT into the model [30]. Higher FEM is not only associated with reduced mortality, but also with a slower progression in end-stage renal disease (ESRD), particularly in the elderly [36, 37]. Even muscle quality seems to play a role [38]. Some studies explained the positive effect of FEM on mortality by the ability of the skeletal muscle cells to produce myokines, which could improve fat oxidation, promote insulin sensitivity and have an anti-inflammatory effect [39–41]. However, sarcopenia is a risk factor for eGFR decline in patients with diabetes, as sarcopenic patients were twice as likely to reach ESRD than those without [36]. Thus, physical activity is recommended for delaying progression of CKD [42]. Nevertheless, it may appear counterintuitive that increased muscle mass is associated with a better eGFR in most studies, because greater muscle mass leads to higher creatinine levels. However, we assume that the beneficial effect of greater muscle mass on general health outweighs this effect. In our study, higher FFMI and BCMI were associated with lower eGFR across both creatinine and Cystatin C-based equations, but, consistent with the aforementioned studies, increased FFM% was associated with a higher eGFR. Since FFM% reflects the proportion of fat mass relative to muscle mass, it may be a surrogate marker for a healthier body composition.

In our study, apart from FFM%, most body composition markers showed a positive association in younger age-groups but an inverse association with eGFR in the age groups from middle adulthood onward. This could be explained by compensatory hyperfiltration due to increased body mass in younger age-groups. Bosma *et al*. found a higher BMI associated with increased glomerular pressure and higher eGFR in young, non-obese individuals [12]. In line with the Brenner hypothesis, this compensatory mechanism may ultimately fail in the future and lead to a decrease in eGFR, especially with comorbidities superimposed [43]. Accordingly, we observe an increasing association strength between higher obesity-related parameters e.g. BMI and lower eGFR in individuals in late adolescence or older.

Cystatin C concentrations are dependent on smoking, thyroid function, chronic inflammation with insulin resistance or elevated C-reactive protein and *higher* adiposity [44]. We adjusted for all of these factors, apart from obesity. With median BMI being within the pre-obesity range, the number of individuals with severe adiposity was limited. Even after additional adjustment for BMI, higher body or fat mass was mostly associated with lower eGFR in females, but associations between anthropometric markers and eGFR were no longer observed. The effect of age remained mostly unchanged (Supplementary Materials). Therefore, while the influence of higher adiposity on Cystatin C-based equations is evident, it is limited. However, even for CKD-EPI_crea+cys_ or EKFC_crea+cys_ most of the potential bias is likely attributable to creatinine. In healthy populations, both endogenous markers are expected to provide comparable estimates of eGFR [45].

Consequently, there was only a small difference in effect sizes of variables representing muscle mass (BCMI, FFMI) between creatinine- and Cystatin C-based equations. KDIGO recommends using both parameters in cases of advanced diseases, as eGFR obtained from the two equations may differ significantly because of sarcopenia [44]. This so-called eGFR difference can predict morbidity and mortality in serious diseases, but is usually greater than 10 ml/min/1.73 m² in this case [46, 47]. Our healthy study population does not suffer from wasting conditions; hence the relatively small difference may be explained by that. Additionally, female sex is associated with a greater eGFR difference. When both sexes are considered separately, women show a larger eGFR difference in our study.

### Limitations

In our study eGFR was only estimated rather than directly measured, since clearance measurement of an exogenous filtration marker was unavailable. The eGFR in our study also fell into the range affected by estimation inaccuracies with calculations for high eGFR values (>60 ml/min/1.73 m^2^), which we compensated for by using several recommended equations. Additionally, selection bias may have arisen as health-conscious and mobile individuals may have been more likely to participate. This may limit the generalizability of the results to the general population or individuals with CKD.

### Conclusion

While higher absolute body mass was associated with lower eGFR, an increased relative FEM was associated with an elevated eGFR, independent of the method used. Additionally, we could show that these associations were more pronounced in women and older individuals. In summary, the results highlight the importance of body composition, sex and age for the glomerular filtration rate. Further research is required to determine the mechanisms underlying the observed differences between sexes and age groups.

## Supplementary Material

sfag261_Supplemental_File

## Data Availability

The data from the Study of Health in Pomerania is available after reasoned request and with the resulting permission through the FVCM Transfer Unit for Data and Biomaterials from the University Medicine Greifswald, Germany.
