# Supplementary material for: Sex- and age-specific associations between body composition markers and estimated glomerular filtration rate–results of a population-based study
Source: Clin Kidney J. 2026 Aug 7;19(9):sfag261. doi: 10.1093/ckj/sfag261 (PMC13548852; doi:10.1093/ckj/sfag261)
Supplement: Supplementary file 1 [file sfag261_supplemental_file.docx]

**Supplementary Material Table 1.** Equations for eGFR in ml/min/1,73m²

| Specification | Serum Creatinine  (µmol/l) | Serum Cystatin C  (mg/l) | Equation for eGFR |
| --- | --- | --- | --- |
| CKD-EPI Creatinine 2009 | | | |
| Female | ≤62 |  | 144×((CREA/88.4)/0.7)^−0.329^ × 0.993^Age^[×1.15][^a^](https://pmc.ncbi.nlm.nih.gov/articles/PMC12929174/#Tfn2) |
|  | >62 |  | 144×((CREA/88.4)/0.7)^−1.209^ × 0.993 ^Age^ [×1.15][^a^](https://pmc.ncbi.nlm.nih.gov/articles/PMC12929174/#Tfn2) |
| Male | ≤80 |  | 141×((CREA/88.4)/0.9)^−0.411^ × 0.993 ^Age^ [×1.14][^a^](https://pmc.ncbi.nlm.nih.gov/articles/PMC12929174/#Tfn2) |
|  | >80 |  | 141×((CREA/88.4)/0.9)^−1.209^ × 0.993 ^Age^ [×1.14][^a^](https://pmc.ncbi.nlm.nih.gov/articles/PMC12929174/#Tfn2) |
| CKD-EPI Creatinine + Cystatin C 2012 | | | |
| Female | ≤62 | ≤ 0.8  > 0.8 | 130×((CREA/88.4)/0.7)^−0.248^×(Cys C/0.8)^−0.375^ × 0.995^Age^[×1.08][^a^](https://pmc.ncbi.nlm.nih.gov/articles/PMC12929174/#Tfn2)  130×((CREA/88.4)/0.7)^−0.248^×(Cys C/0.8)^−0.711^ × 0.995^Age^[×1.08][^a^](https://pmc.ncbi.nlm.nih.gov/articles/PMC12929174/#Tfn2) |
|  | >62 | ≤ 0.8  > 0.8 | 130×((CREA/88.4)/0.7)^−0.601^×(Cys C/0.8)^−0.375^ × 0.995^Age^[×1.08][^a^](https://pmc.ncbi.nlm.nih.gov/articles/PMC12929174/#Tfn2)  130×((CREA/88.4)/0.7)^−0.601^×(Cys C/0.8)^−0.711^ × 0.995^Age^[×1.08][^a^](https://pmc.ncbi.nlm.nih.gov/articles/PMC12929174/#Tfn2) |
| Male | ≤80 | ≤ 0.8  > 0.8 | 135×((CREA/88.4)/0.9)^−0.207^×(Cys C/0.8)^−0.375^ × 0.995^Age^[×1.08][^a^](https://pmc.ncbi.nlm.nih.gov/articles/PMC12929174/#Tfn2)  135×((CREA/88.4)/0.9)^−0.207^×(Cys C/0.8)^−0.711^ × 0.995^Age^[×1.08][^a^](https://pmc.ncbi.nlm.nih.gov/articles/PMC12929174/#Tfn2) |
|  | >80 | ≤ 0.8  > 0.8 | 135×((CREA/88.4)/0.9)^−0.601^×(Cys C/0.8)^−0.375^ × 0.995^Age^[×1.08][^a^](https://pmc.ncbi.nlm.nih.gov/articles/PMC12929174/#Tfn2)  135×((CREA/88.4)/0.9)^−0.601^×(Cys C/0.8)^−0.711^ × 0.995^Age^[×1.08][^a^](https://pmc.ncbi.nlm.nih.gov/articles/PMC12929174/#Tfn2) |
| EKFC Creatinine 2021 | | | |
| Female & Male | cr/Q* ≤1 |  | 107.3/(CREA/Q*)^0.322^×[0.99^Age−40^]^b^ |
|  | cr/Q* >1 |  | 107.3/(CREA/Q*)^1.132^×[0.99^Age−40^]^b^ |
| * Q = calculated as follows:  For ages 2–25 years: Women: ln(Q) = 3.080 + 0.177 × Age − 0.223 × ln(Age) − 0.00596 × Age^2^ + 0.0000686 × Age^3^.  Men: ln(Q) = 3.200 + 0.259 × Age − 0.543 × ln(Age) − 0.00763 × Age^2^ + 0.0000790 × Age^3^.  For ages >25 years: Women: Q = 62 μmol/L, Men Q = 80 μmol/L. | | | |
| EKFC Cystatin C 2023 | | | |
| Female & Male |  | cys/Q^†^ ≤1 | 107.3/(CYS/Q^†^)^0.322^×[0.99^Age−40^]^b^ |
|  |  | cys/Q^†^ >1 | 107.3/(CYS/Q^†^)^1.132^×[0.99^Age−40^]^b^ |
| ^†^ Q = calculated as follows:  For ages <50 years: Women & men: Q = 0.83 mg/L. For ages >50 years, Q = 0.83 + 0.005*(Age-50) mg/L. | | | |
| EKFC Creatinine + Cystatin C | | | |
| Female & Male |  |  | (eGFR(EKFC CREA 2021)+eGFR(EKFC Cys C 2023))/2 |

^a^ = Factor to apply if the patient is African American; ^b^ Factor to apply if age >40 years. eGFR = estimated glomerular filtration rate, CKD-EPI = Chronic Kidney Disease Epidemiology Collaboration, EKFC = European Kidney Function Consortium, crea = serum creatinine, cys C = serum Cystatin C, Q = rescaling factor

| **Supplementary Material Table 2.** Complete characteristics of the study population stratified by sex-specific quartiles of waist circumference | | | | | | |
| --- | --- | --- | --- | --- | --- | --- |
|  | **Waist circumference** | | | | | |
|  | **Quartile 1** | **Quartile 2** | **Quartile 3** | **Quartile 4** | **p*** |  |
| **N** | 1,099 (25.3%) | 1,079 (24.9%) | 1,090 (25.1%) | 1,069 (24.6%) |  |  |
| **Age in years** | 39 (30; 49) | 51 (40; 61) | 58 (46; 68) | 60 (51; 69) | <0.001 |  |
| **Sex** |  |  |  |  |  |  |
| Male | 535 (48.7%) | 519 (48.1%) | 525 (48.2%) | 525 (49.1%) | 0.962 |  |
| Female | 564 (51.3%) | 560 (51.9%) | 565 (51.8%) | 544 (50.9%) |  |  |
| **Years of education** |  |  |  |  |  |  |
| less than 10 years | 135 (12.3%) | 183 (17.0%) | 299 (27.4%) | 389 (36.4%) | <0.001 |  |
| 10 years | 582 (53.0%) | 603 (55.9%) | 550 (50.5%) | 502 (47.0%) |  |  |
| more than 10 years | 382 (34.8%) | 293 (27.2%) | 241 (22.1%) | 178 (16.7%) |  |  |
| **Smoking status** |  |  |  |  |  |  |
| Never smokers | 387 (35.2%) | 391 (36.2%) | 423 (38.8%) | 380 (35.5%) | <0.001 |  |
| Former smokers | 320 (29.1%) | 371 (34.4%) | 420 (38.5%) | 481 (45.0%) |  |  |
| Current smokers | 392 (35.7%) | 317 (29.4%) | 247 (22.7%) | 208 (19.5%) |  |  |
| **Physically active** | 794 (72.2%) | 798 (74.0%) | 732 (67.2%) | 673 (63.1%) | <0.001 |  |
| **Alcohol consumption; g/day** | 3.6 (1.0; 10.6) | 4.3 (1.3; 12.0) | 3.4 (0.7; 10.8) | 2.1 (0.3; 8.7) | 0.052 |  |
| **Food frequency score** | 13.0 (11.0; 16.0) | 14.0 (12.0; 16.0) | 14.0 (12.0; 16.0) | 14.0 (12.0; 17.0) | <0.001 |  |
| **Body height; cm** | 171 (164; 178) | 170 (164; 177) | 169 (162; 176) | 169 (162; 176) | <0.001 |  |
| **Body weight; kg** | 66 (58; 76) | 75 (67; 83) | 84 (75; 92) | 97 (88; 107) | <0.001 |  |
| **Body surface area; m²** | 1.77 (1.64; 1.93) | 1.86 (1.74; 2.00) | 1.95 (1.81; 2.08) | 2.07 (1.93; 2.22) | <0.001 |  |
| **Body mass index; kg/m²** | 22.8 (21.2; 24.7) | 26.0 (24.5; 27.5) | 29.1 (27.6; 30.7) | 33.6 (31.4; 36.8) | <0.001 |  |
| **Waist circumference; cm** | 74 (70; 84) | 83 (79; 92) | 94 (88; 101) | 108 (102; 114) | <0.001 |  |
| **Waist-to-hip ratio; %** | 82 (77; 87) | 87 (81; 94) | 91 (84; 97) | 95 (88; 102) | <0.001 |  |
| **Fat mass; kg** | 15.2 (12.4; 17.9) | 20.3 (17.6; 23.5) | 25.5 (21.9; 29.3) | 33.3 (28.5; 39.7) | <0.001 |  |
| **Fat mass index; kg/m²** | 5.2 (4.3; 6.2) | 7.0 (5.9; 8.3) | 8.8 (7.5; 10.6) | 11.9 (9.7; 14.6) | <0.001 |  |
| **Fat-free mass; kg** | 49.9 (43.4; 60.7) | 52.4 (45.8; 64.2) | 56.1 (48.1; 68.1) | 61.5 (52.3; 73.5) | <0.001 |  |
| **Fat-free mass index; kg/m²** | 17.4 (15.9; 19.2) | 18.5 (16.9; 20.7) | 19.9 (18.1; 22.1) | 21.7 (19.8; 23.9) | <0.001 |  |
| **Body cell mass; kg** | 26.0 (22.2; 33.9) | 27.2 (23.6; 34.6) | 28.7 (24.2; 36.6) | 31.0 (26.0; 38.8) | <0.001 |  |
| **Body cell mass index; kg/m²** | 9.1 (8.1; 10.7) | 9.7 (8.7; 11.0) | 10.2 (9.1; 11.7) | 11.1 (9.9; 12.6) | <0.001 |  |
| **Relative fat-free mass; %** | 77 (73; 81) | 72 (68; 77) | 69 (63; 74) | 64 (58; 71) | <0.001 |  |
| **Subcutaneous fat; L** | 4.57 (3.74; 5.72) | 6.91 (5.75; 7.99) | 8.96 (7.48; 10.7) | 12.0 (9.72; 14.7) | <0.001 |  |
| **Visceral fat; L** | 1.43 (0.82; 2.52) | 3.04 (1.94; 4.58) | 4.79 (3.37; 6.54) | 6.66 (5.02; 8.59) | <0.001 |  |
| **Liver fat; %** | 2.3 (1.9; 3.1) | 3.4 (2.3; 5.6) | 5.3 (3.3; 10.1) | 9.7 (5.8; 17.4) | <0.001 |  |
| **HbA1c; %** | 5.0 (4.7; 5.4) | 5.2 (4.8; 5.5) | 5.3 (5.0; 5.7) | 5.6 (5.2; 6.1) | <0.001 |  |
| **Type 2 diabetes** | 33 (3.0%) | 71 (6.6%) | 134 (12.3%) | 301 (28.2%) | <0.001 |  |
| **Total cholesterol; mmol/L** | 5.1 (4.4; 5.9) | 5.4 (4.7; 6.2) | 5.6 (4.8; 6.3) | 5.4 (4.7; 6.2) | <0.001 |  |
| **HDL-cholesterol; mmol/L** | 1.52 (1.27; 1.81) | 1.47 (1.21; 1.75) | 1.35 (1.14; 1.60) | 1.24 (1.05; 1.49) | <0.001 |  |
| **LDL-cholesterol; mmol/L** | 3.0 (2.5; 3.7) | 3.3 (2.7; 3.9) | 3.4 (2.8; 4.1) | 3.5 (2.8; 4.0) | <0.001 |  |
| **Triglycerides; mmol/L** | 1.0 (0.7; 1.4) | 1.2 (0.9; 1.8) | 1.6 (1.1; 2.2) | 1.8 (1.3; 2.6) | <0.001 |  |
| **Lipid-lowering medication intake** | 32 (2.9%) | 104 (9.6%) | 200 (18.3%) | 231 (21.6%) | <0.001 |  |
| **Systolic blood pressure; mmHg** | 118 (108; 129) | 125 (114; 137) | 130 (120; 142) | 134 (122; 146) | <0.001 |  |
| **Diastolic blood pressure; mmHg** | 72 (66; 79) | 76 (70; 82) | 78 (72; 85) | 80 (74; 86) | <0.001 |  |
| **Intake of antihypertensive medication** | 118 (10.7%) | 286 (26.5%) | 464 (42.6%) | 639 (59.8%) | <0.001 |  |
| **Hypertension** | 222 (20.2%) | 418 (38.8%) | 646 (59.3%) | 804 (75.5%) | <0.001 |  |
| **C-reactive protein; mg/L** | 0.69 (0.37; 1.44) | 1.05 (0.58; 2.23) | 1.45 (0.82; 2.74) | 2.65 (1.35; 5.14) | <0.001 |  |
| **Creatinine; µmol/L** | 74 (65; 85) | 75 (65; 86) | 78 (67; 90) | 80 (70; 93) | <0.001 |  |
| **Cystatin C; mg/L** | 0.66 (0.60; 0.74) | 0.69 (0.62; 0.76) | 0.73 (0.66; 0.83) | 0.78 (0.69; 0.90) | <0.001 |  |
| **eGFR (EKFC_crea_); ml/min/1.73 m²** | 95 (84; 106) | 89 (74; 100) | 81 (68; 93) | 77 (63; 90) | <0.001 |  |
| **Low eGFR (EKFC_crea_)** | 32 (2.9%) | 86 (8.0%) | 169 (15.5%) | 220 (20.6%) | <0.001 |  |
| **eGFR (EKFC_cys_); ml/min/1.73 m²** | 117 (103; 128) | 104 (92; 117) | 95 (84; 108) | 91 (80; 102) | <0.001 |  |
| **Low eGFR (EKFC_cys_)** | 3 (0.3%) | 13 (1.2%) | 28 (2.6%) | 62 (5.8%) | <0.001 |  |
| **eGFR (EKFC_crea+cys_); ml/min/1.73 m²** | 107 (96; 115) | 96 (84; 108) | 89 (76; 100) | 84 (73; 96) | <0.001 |  |
| **Low eGFR (EKFC_crea+cys_)** | 5 (0.5%) | 29 (2.7%) | 58 (5.3%) | 108 (10.1%) | <0.001 |  |
| **eGFR (CKD-EPI_crea_); ml/min/1.73 m²** | 99 (87; 111) | 92 (78; 104) | 86 (72; 98) | 81 (67; 95) | <0.001 |  |
| **Low eGFR (CKD-EPI_crea_)** | 22 (2.0%) | 54 (5.0%) | 115 (10.6%) | 170 (15.9%) | <0.001 |  |
| **eGFR (CKD-Epi_crea+cys_); ml/min/1.73 m²** | 112 (101; 121) | 105 (93; 116) | 97 (84; 109) | 93 (78; 105) | <0.001 |  |
| **Low eGFR (CKD-Epi_crea+cys_)** | 6 (0.5%) | 25 (2.3%) | 53 (4.9%) | 103 (9.6%) | <0.001 |  |
| **ACR; mg/g** | 93 (8.5%) | 89 (8.3%) | 150 (13.8%) | 204 (19.1%) | <0.001 |  |

Data are expressed as median, 25^th^, and 75^th^ percentile (continuous data) or as absolute numbers and percentages (categorical data). Exposure variables were standardised per standard deviation. ACR = urinary albumin creatinine ratio, BMI = body mass index, eGFR = estimated glomerular filtration rate, EKFC = European Kidney Function Consortia equation; CKD-EPI = Chronic Kidney Disease Epidemiology Collaboration equation, crea = creatinine-based formula; cys = Cystatin C- based formula, crea+ cys = Cystatin C-and creatinine-based formula, HbA1c = glycosylated haemoglobin, HDL-Cholesterol = high-density lipoprotein cholesterol, LDL-Cholesterol = low-density lipoprotein cholesterol, * p-values derived from Kruskal-Walllis-tests (continuous data) or χ2-tests (categorical data)

**Supplementary Material Table 3.** Associations of body composition markers with estimated glomerular filtration rate (eGFR)

|  | **EKFC_crea_**  β (95%-CI) | **EKFC_cys_**  β (95%-CI) | **EKFC_crea+cys_**  β (95%-CI) | **CKD-Epi_crea_**  β (95%-CI) | **CKD-Epi_crea+cys_**  β (95%-CI) |
| --- | --- | --- | --- | --- | --- |
| **Body surface area; m^2^** | -1.01 (-1.51; -0.51)* | -1.50 (-1.79; -1.20)* | -1.25 (-1.59; -0.91)* | -1.60 (-2.14; -1.05)* | -2.11 (-2.64; -1.59)* |
| **Body mass index; kg/m^2^** | -0.69 (-1.12; -0.25)* | -1.31 (-1.56; -1.04)* | -1.00 (-1.29; -0.70)* | -1.06 (-1.53; -0.59)* | -1.65 (-2.10; -1.19)* |
| **Waist circumference; cm** | -0.49 (-0.99; 0.00) | -1.59 (-1.89; -1.30)* | -1.04 (-1.38; -0.70)* | -0.94 (-1.48; -0.39)* | -1.82 (-2.35; -1.30)* |
| **Waist-to-hip ratio** | 0.16 (-0.43; 0.74) | -0.77 (-1.12; -0.42)* | -0.31 (-0.71; 0.10) | -0.33 (-0.97; 0.31) | -0.58 (-1.20; 0.04) |
| **Fat mass index; kg/m2** | -0.52 (-0.99; -0.06)* | -1.50 (-1.78; -1.23)* | -1.01 (-1.33; -0.70)* | -0.78 (-1.29; -0.27)* | -1.76 (-2.24; -1.27)* |
| **Fat-free mass index; kg/m2** | -1.20 (-1.75; -0.65)* | -1.06 (-1.39; -0.73)* | -1.13 (-1.51; -0.76)* | -1.82 (-2.42; -1.22)* | -1.75 (-2.33; -1.17)* |
| **Body cell mass index; kg/m2** | -1.31 (-1.84; -0.77)* | -0.51 (-0.82; -0.19)* | -0.91 (-1.27; -0.54)* | -2.27 (-2.85; -1.69)* | -1.20 (-1.76; -0.64)* |
| **Relative fat-free mass; %** | 0.11 (-0.44; 0.65) | 1.46 (1.13; 1.78)* | 0.78 (0.41; 1.15)* | 0.25 (-0.34; 0.85) | 1.47 (0.89; 2.04)* |
| **Subcutaneous fat, L** | 0.01 (-0.60; 0.62) | -1.23 (-1.57; -0.90)* | -0.61 (-1.02; -0.21)* | -0.22 (-0.89; 0.44) | -1.28 (-1.91; -0.64)* |
| **Visceral fat; L** | -0.65 (-1.40; 0.10) | -1.07 (-1.49; -0.65)* | -0.86 (-1.36; -0.36)* | -0.91 (-1.73; -0.09)* | -1.51 (-2.29; -0.73)* |
| **Liver fat; %** | -0.90 (-1.49; -0.31)* | -0.80 (-1.12; -0.48)* | -0.85 (-1.24; -0.46)* | -1.14 (-1.79; -0.50)* | -1.28 (-1.90; -0.67)* |

Results are reported as standardised beta coefficients derived from linear regression models adjusted for age, sex, smoking status, years of education, diabetes, fasting glucose, alcohol, physical activity, dietary intake and C-reactive protein. Exposure variables were standardised per standard deviation. EKFC = European Kidney Function Consortia equation; CKD-EPI = Chronic Kidney Disease Epidemiology Collaboration equation, crea = creatinine-based formula; cys = Cystatin C- based formula, crea+ cys = Cystatin C-and creatinine-based formula, CI = confidence interval, *p<0.05

| **Supplementary Material Table 4.** Characteristics of the study population stratified by sex | | | |
| --- | --- | --- | --- |
|  | **Male** | **Female** | **p*** |
| **N** | 2,104 (48.5%) | 2,233 (51.5%) |  |
| **Age in years** | 53 (40; 65) | 52 (39; 63) | 0.011 |
| **Years of education** |  |  | <0.001 |
| less than 10 years | 521 (24.8%) | 485 (21.7%) |  |
| 10 years | 992 (47.1%) | 1,245 (55.8%) |  |
| more than 10 years | 591 (28.1%) | 503 (22.5%) |  |
| **Smoking status** |  |  | <0.001 |
| Never smokers | 540 (25.7%) | 1,041 (46.6%) |  |
| Former smokers | 952 (45.2%) | 640 (28.7%) |  |
| Current smokers | 612 (29.1%) | 552 (24.7%) |  |
| **Physically active** | 1,447 (68.8%) | 1,550 (69.4%) | 0.683 |
| **Alcohol consumption; g/day** | 7.8 (2.0; 18.5) | 1.8 (0.4; 4.9) | <0.001 |
| **Food frequency score** | 13.0 (11.0; 15.0) | 15.0 (13.0; 17.0) | <0.001 |
| **Body height; cm** | 176 (172; 181) | 164 (159; 168) | <0.001 |
| **Body weight; kg** | 87 (78; 98) | 72 (63; 82) | <0.001 |
| **Body surface area; m²** | 2.04 (1.93; 2.16) | 1.78 (1.68; 1.90) | <0.001 |
| **Body mass index; kg/m²** | 28.2 (25.5; 31.2) | 26.7 (23.4; 30.9) | <0.001 |
| **Waist circumference; cm** | 97 (88; 106) | 84 (75; 94) | <0.001 |
| **Waist-to-hip ratio; %** | 95 (90; 100) | 82 (78; 87) | <0.001 |
| **Fat mass; kg** | 20.8 (16.3; 26.1) | 24.5 (18.4; 32.1) | <0.001 |
| **Fat mass index; kg/m²** | 6.7 (5.3; 8.4) | 9.1 (6.8; 12.0) | <0.001 |
| **Fat-free mass; kg** | 66.6 (61.1; 72.5) | 47.2 (43.9; 51.3) | <0.001 |
| **Fat-free mass index; kg/m²** | 21.4 (19.8; 23.0) | 17.6 (16.4; 19.0) | <0.001 |
| **Body cell mass; kg** | 35.9 (32.3; 39.5) | 24.1 (21.9; 26.4) | <0.001 |
| **Body cell mass index; kg/m²** | 11.5 (10.5; 12.6) | 8.9 (8.2; 9.8) | <0.001 |
| **Relative fat-free mass; %** | 76 (72; 80) | 66 (61; 71) | <0.001 |
| **Subcutaneous fat; L** | 6.51 (4.91; 8.53) | 8.31 (6.19; 11.13) | <0.001 |
| **Visceral fat; L** | 5.16 (3.12; 7.13) | 2.49 (1.27; 3.95) | <0.001 |
| **Liver fat; %** | 4.8 (2.8; 9.1) | 3.2 (2.1; 6.8) | <0.001 |
| **HbA1c; %** | 5.3 (5.0; 5.8) | 5.2 (4.8; 5.6) | <0.001 |
| **Type 2 diabetes** | 304 (14.4%) | 235 (10.5%) | <0.001 |
| **Total cholesterol; mmol/L** | 5.3 (4.5; 6.0) | 5.5 (4.8; 6.3) | <0.001 |
| **HDL-cholesterol; mmol/L** | 1.24 (1.05; 1.45) | 1.56 (1.32; 1.81) | <0.001 |
| **LDL-cholesterol; mmol/L** | 3.3 (2.6; 3.9) | 3.3 (2.7; 4.0) | 0.069 |
| **Triglycerides; mmol/L** | 1.5 (1.1; 2.3) | 1.3 (0.9; 1.8) | <0.001 |
| **Lipid-lowering medication intake** | 333 (15.8%) | 234 (10.5%) | <0.001 |
| **Systolic blood pressure; mmHg** | 132 (124; 144) | 119 (109; 132) | <0.001 |
| **Diastolic blood pressure; mmHg** | 80 (72; 86) | 74 (68; 81) | <0.001 |
| **Intake of antihypertensive medication** | 769 (36.6%) | 738 (33.0%) | 0.015 |
| **Hypertension** | 1,177 (56.0%) | 913 (40.9%) | <0.001 |
| **C-reactive protein; mg/L** | 1.15 (0.60; 2.48) | 1.44 (0.72; 3.38) | <0.001 |
| **Creatinine; µmol/L** | 85 (76; 95) | 69 (61; 77) | <0.001 |
| **Cystatin C; mg/L** | 0.73 (0.66; 0.82) | 0.69 (0.62; 0.78) | <0.001 |
| **eGFR (EKFC_crea_); ml/min/1.73 m²** | 87 (73; 99) | 85 (71; 97) | 0.006 |
| **Low eGFR (EKFC_crea_)** | 215 (10.2%) | 292 (13.1%) | 0.003 |
| **eGFR (EKFC_cys_); ml/min/1.73 m²** | 99 (86; 114) | 102 (89; 119) | <0.001 |
| **Low eGFR (EKFC_cys_)** | 60 (2.9%) | 46 (2.1%) | 0.092 |
| **eGFR (EKFC_crea+cys_); ml/min/1.73 m²** | 94 (80; 106) | 95 (81; 107) | 0.078 |
| **Low eGFR (EKFC_crea+cys_)** | 100 (4.8%) | 100 (4.5%) | 0.667 |
| **eGFR (CKD-EPI_crea_); ml/min/1.73 m²** | 90 (76; 103) | 90 (75; 102) | 0.485 |
| **Low eGFR (CKD-Epi_crea_)** | 160 (7.6%) | 201 (9.0%) | 0.096 |
| **eGFR (CKD-Epi_crea+cys_); ml/min/1.73 m²** | 104 (90; 115) | 101 (87; 113) | 0.002 |
| **Low eGFR (CKD-Epi_crea+cys_)** | 91 (4.3%) | 96 (4.3%) | 0.966 |
| **ACR; mg/g** | 238 (11.3%) | 298 (13.4%) | 0.043 |

Data are expressed as median, 25^th^, and 75^th^ percentile (continuous data) or as absolute numbers and percentages (categorical data). Exposure variables were standardised per standard deviation. ACR = urinary albumin creatinine ratio, BMI = body mass index, eGFR = estimated glomerular filtration rate, EKFC = European Kidney Function Consortia equation; CKD-EPI = Chronic Kidney Disease Epidemiology Collaboration equation, crea = creatinine-based formula; cys = Cystatin C- based formula, crea+ cys = Cystatin C-and creatinine-based formula, HbA1c = glycosylated haemoglobin, HDL-Cholesterol = high-density lipoprotein cholesterol, LDL-Cholesterol = low-density lipoprotein cholesterol, * p-values derived from Kruskal-Walllis-tests (continuous data) or χ2-tests (categorical data)

**Supplementary Material Table 5.** Associations of body composition markers with estimated glomerular filtration rate (eGFR) in females

|  | **EKFC_crea_**  β (95%-CI) | **EKFC_cys_**  β (95%-CI) | **EKFC_crea+cys_**  β (95%-CI) | **CKD-Epi_crea_**  β (95%-CI) | **CKD-Epi_crea+cys_**  β (95%-CI) |
| --- | --- | --- | --- | --- | --- |
| **Body surface area; m^2^** | -1.47 (-2.21; -0.73)* | -1.78 (-2.18; -1.38)* | -1.62 (-2.11; -1.14)* | -1.96 (-2.77; -1.16)* | -2.79 (-3.53; -2.05)* |
| **Body mass index; kg/m^2^** | -1.02 (-1.61; -0.44)* | -1.46 (-1.77; -1.15)* | -1.24 (-1.62; -0.86)* | -1.29 (-1.93; -0.66)* | -2.07 (-2.66; -1.49)* |
| **Waist circumference; cm** | -1.04 (-1.74; -0.34)* | -1.87 (-2.24; -1.49)* | -1.45 (-1.92; -0.99)* | -1.39 (-2.15; -0.62)* | -2.52 (-3.23; -1.81)* |
| **Waist-to-hip ratio** | -0.40 (-1.28; 0.48) | -1.00 (-1.48; -0.52)* | -0.70 (-1.28; -0.11)* | -0.76 (-1.72; 0.20) | -1.22 (-2.12; -0.33)* |
| **Fat mass index; kg/m2** | -0.87 (-1.45; -0.29)* | -1.59 (-1.90; -1.28)* | -1.23 (-1.61; -0.85)* | -1.05 (-1.68; -0.42)* | -2.07 (-2.66; -1.49)* |
| **Fat-free mass index; kg/m2** | -2.03 (-2.94; -1.12)* | -1.58 (-2.07; -1.08)* | -1.80 (-2.40; -1.20)* | -2.65 (-3.63; -1.67)* | -2.91 (-3.82; -1.99)* |
| **Body cell mass index; kg/m2** | -2.51 (-3.39; -1.62)* | -1.21 (-1.70; -0.73)* | -1.86 (-2.44; -1.27)* | -3.57 (-4.53; -2.62)* | -2.85 (-3.74; -1.95)* |
| **Relative fat-free mass; %** | 0.54 (-0.18; 1.27) | 1.72 (1.33; 2.11)* | 1.13 (0.65; 1.61)* | 0.60 (-0.19; 1.38) | 1.98 (1.25; 2.71)* |
| **Subcutaneous fat; L** | -0.09 (-0.91; 0.73) | -1.29 (-1.73; -0.85)* | -0.69 (-1.23; -0.15)* | -0.14 (-1.03; 0.75) | -1.36 (-2.18; -0.53)* |
| **Visceral fat; L** | -1.61 (-3.06; -0.18)* | -2.44 (-3.21; -1.66)* | -2.03 (-2.97; -1.09)* | -1.62 (-3.19; -0.06)* | -3.43 (-4.88; -1.98)* |
| **Liver fat; %** | -1.55 (-2.45; -0.64)* | -1.15 (-1.62; -0.68)* | -1.35 (-1.93; -0.77)* | -1.58 (-2.57; -0.60)* | -2.10 (-3.01; -1.19)* |

Results are reported as standardised beta coefficients derived from linear regression models for age, smoking status, years of education, diabetes, fasting glucose, alcohol, physical activity, dietary intake and C-reactive protein. Exposure variables were standardised per standard deviation. EKFC = European Kidney Function Consortia equation; CKD-EPI = Chronic Kidney Disease Epidemiology Collaboration equation, crea = serum creatinine, cys = serum Cystatin C, crea+cys = Cystatin C + creatinine, CI = confidence interval, *p<0.05

**Supplementary Material Table 6.** Associations of body composition markers with estimated glomerular filtration rate (eGFR) in males

|  | **EKFC_crea_**  β (95%-CI) | **EKFC_cys_**  β (95%-CI) | **EKFC_crea+cys_**  β (95%-CI) | **CKD-Epi_crea_**  β (95%-CI) | **CKD-Epi_crea+cys_**  β (95%-CI) |
| --- | --- | --- | --- | --- | --- |
| **Body surface area; m^2^** | -0.58(-1.25; 0.10) | -1.20 (-1.65; -0.76)* | -0.89 (-1.37; -0.41)* | -1.22 (-1.97; -0.48)* | -1.48 (-2.23; -0.73)* |
| **Body mass index; kg/m^2^** | -0.18 (-0.84; 0.49) | -1.04 (-1.47; -0.60)* | -0.61 (-1.08; -0.13)* | -0.68 (-1.41; 0.05) | -0.99 (-1.73; -0.26)* |
| **Waist circumference; cm** | 0.14 (-0.56; 0.85) | -1.27 (-1.73; -0.81)* | -0.56 (-1.07; -0.06)* | -0.40 (-1.17; 0.38) | -1.02 (-1.80; -0.24)* |
| **Waist-to-hip ratio** | 0.68 (-0.11; 1.46) | -0.70 (-1.21; -0.18)* | -0.01 (-0.57; 0.55) | 0.02 (-0.83; 0.88) | -0.06 (-0.93; 0.80) |
| **Fat mass index; kg/m2** | 0.34 (-0.48; 1.17) | -1.19 (-1.72; -0.66)* | -0.42 (-1.01; 0.16) | -0.04 (-0.94; 0.87) | -0.92 (-1.83; -0.02)* |
| **Fat-free mass index; kg/m2** | -0.65 (-1.33; 0.03) | -0.72 (-1.16; -0.28)* | -0.69 (-1.17; -0.20)* | -1.24 (-1.99; -0.50)* | -0.99 (-1.74; -0.24)* |
| **Body cell mass index; kg/m2** | -0.49 (-1.15; 0.17) | 0.08 (-0.35; 0.50) | -0.21 (-0.68; 0.26) | -1.34 (-2.06; -0.61)* | -0.04 (-0.77; 0.69) |
| **Relative fat-free mass; %** | -0.62 (-1.47; 0.23) | 0.91 (0.36; 1.45)* | 0.14 (-0.46; 0.75) | -0.39 (-1.32; 0.54) | 0.54 (-0.39; 1.48) |
| **Subcutaneous fat; L** | 0.22 (-0.72; 1.18) | -1.05 (-1.59; -0.51)* | -0.41 (-1.05; 0.23) | -0.28 (-1.32; 0.76) | -1.07 (-2.09; -0.05)* |
| **Visceral fat; L** | -0.31 (-1.16; 0.53) | -0.61 (-1.10; -0.13)* | -0.46 (-1.04; 0.11) | -0.70 (-1.63; 0.23) | -0.83 (-1.74; 0.08) |
| **Liver fat; %** | -0.49 (-1.26; 0.29) | -0.46 (-0.91; 0.01) | -0.47 (-1.00; 0.06) | -0.91 (-1.77; -0.06)* | -0.65 (-1.50; 0.19) |

Results are reported as standardised beta coefficients derived from linear regression models adjusted for age, smoking status, years of education, diabetes, fasting glucose, alcohol, physical activity, dietary intake and C-reactive protein. Exposure variables were standardised per standard deviation. EKFC = European Kidney Function Consortia equation; CKD-EPI equation = Chronic Kidney Disease Epidemiology Collaboration equation, crea = serum creatinine, cys = serum Cystatin C, crea+cys = Cystatin C + creatinine, CI = confidence interval, *p<0.05

**Supplementary Figures: Sex-related standardised beta coefficients for various body composition markers and eGFR in different age groups**

**Supplementary Figure 1.** Standardised beta coefficients for BMI and eGFR (EKFC_crea+cys_) in different age groups for both sexes. Results are reported as mean with confidence interval for each age group. Linear regression model adjusted for age, smoking status, years of education, diabetes, fasting glucose, alcohol, physical activity, dietary intake and C-reactive protein, exposure variables standardised per standard deviation. BMI = body mass index; eGFR = estimated glomerular filtration rate; EKFC (CysCrea) = European Kidney Function Consortia equation using Cystatin C + creatinine

**Supplementary Figure 2.** Standardised beta coefficients for waist circumference and eGFR (EKFC_crea+cys_) in different age groups for both sexes. Results are reported as mean with confidence interval for each age group. Linear regression model adjusted for age, smoking status, years of education, diabetes, fasting glucose, alcohol, physical activity, dietary intake and C-reactive protein, exposure variables standardised per standard deviation. eGFR = estimated glomerular filtration rate; EKFC (CysCrea) = European Kidney Function Consortia equation using Cystatin C + creatinine

**Supplementary Figure 3.** Standardised beta coefficients for waist-to-hip ratio and eGFR (EKFC_crea+cys_) in different age groups for both sexes. Results are reported as mean with confidence interval for each age group. Linear regression model adjusted for age, smoking status, years of education, diabetes, fasting glucose, alcohol, physical activity, dietary intake and C-reactive protein, exposure variables standardised per standard deviation. eGFR = estimated glomerular filtration rate; EKFC (CysCrea) = European Kidney Function Consortia equation using Cystatin C + creatinine

**Supplementary Figure 4.** Standardised beta coefficients for body surface area and eGFR (EKFC_crea+cys_) in different age groups for both sexes. Results are reported as mean with confidence interval for each age group. Linear regression model adjusted for age, smoking status, years of education, diabetes, fasting glucose, alcohol, physical activity, dietary intake and C-reactive protein, exposure variables standardised per standard deviation. eGFR = estimated glomerular filtration rate; EKFC (CysCrea) = European Kidney Function Consortia equation using Cystatin C + creatinine

**Supplementary Figure 5.** Standardised beta coefficients for fat mass index and eGFR (EKFC_crea+cys_) in different age groups for both sexes. Results are reported as mean with confidence interval for each age group. Linear regression model adjusted for age, smoking status, years of education, diabetes, fasting glucose, alcohol, physical activity, dietary intake and C-reactive protein, exposure variables standardised per standard deviation. eGFR = estimated glomerular filtration rate; EKFC (CysCrea) = European Kidney Function Consortia equation using Cystatin C + creatinine

**Supplementary Figure 6.** Standardised beta coefficients for fat-free mass index and eGFR (EKFC_crea+cys_) in different age groups for both sexes. Results are reported as mean with confidence interval for each age group. Linear regression model adjusted for age, smoking status, years of education, diabetes, fasting glucose, alcohol, physical activity, dietary intake and C-reactive protein, exposure variables standardised per standard deviation. eGFR = estimated glomerular filtration rate; EKFC (CysCrea) = European Kidney Function Consortia equation using Cystatin C + creatinine

**Supplementary Figure 7.** Standardised beta coefficients for body cell mass index and eGFR (EKFC_crea+cys_) in different age groups for both sexes. Results are reported as mean with confidence interval for each age group. Linear regression model adjusted for age, smoking status, years of education, diabetes, fasting glucose, alcohol, physical activity, dietary intake and C-reactive protein, exposure variables standardised per standard deviation. eGFR = estimated glomerular filtration rate; EKFC (CysCrea) = European Kidney Function Consortia equation using Cystatin C + creatinine

**Supplementary Figure 8.** Standardised beta coefficients for relative fat-free mass and eGFR (EKFC_crea+cys_) in different age groups for both sexes. Results are reported as mean with confidence interval for each age group. Linear regression model adjusted for age, smoking status, years of education, diabetes, fasting glucose, alcohol, physical activity, dietary intake and C-reactive protein, exposure variables standardised per standard deviation. eGFR = estimated glomerular filtration rate; EKFC (CysCrea) = European Kidney Function Consortia equation using Cystatin C + creatinine

**Supplementary Figure 9.** Standardised beta coefficients for subcutaneous fat and eGFR (EKFC_crea+cys_) in different age groups for both sexes. Results are reported as mean with confidence interval for each age group. Linear regression model adjusted for age, smoking status, years of education, diabetes, fasting glucose, alcohol, physical activity, dietary intake and C-reactive protein, exposure variables standardised per standard deviation. eGFR = estimated glomerular filtration rate; EKFC (CysCrea) = European Kidney Function Consortia equation using Cystatin C + creatinine

**Supplementary Figure 10.** Standardised beta coefficients for visceral fat and eGFR (EKFC_crea+cys_) in different age groups for both sexes. Results are reported as mean with confidence interval for each age group. Linear regression model adjusted for age, smoking status, years of education, diabetes, fasting glucose, alcohol, physical activity, dietary intake and C-reactive protein, exposure variables standardised per standard deviation. eGFR = estimated glomerular filtration rate; EKFC (CysCrea) = European Kidney Function Consortia equation using Cystatin C + creatinine

**Supplementary Figure 11.** Standardised beta coefficients for liver fat and eGFR (EKFC_crea+cys_) in different age groups for both sexes. Results are reported as mean with confidence interval for each age group. Linear regression model adjusted for age, smoking status, years of education, diabetes, fasting glucose, alcohol, physical activity, dietary intake and C-reactive protein, exposure variables standardised per standard deviation. eGFR = estimated glomerular filtration rate; EKFC (CysCrea) = European Kidney Function Consortia equation using Cystatin C + creatinine

**Supplementary Material Table 7.** Associations of body composition markers with estimated glomerular filtration rate (eGFR) excluding eGFR values > 90ml/min/1.73m² (EKFC_crea+cys_)

|  | **Total**  β (95%-CI) | **Females**  β (95%-CI) | **Males**  β (95%-CI) |
| --- | --- | --- | --- |
| **Body surface area; m^2^** | -1.19 (-1.79; -0.59)* | -1.91 (-2.75; -1.07)* | -0.57 (-1.42; 0.29) |
| **Body mass index; kg/m^2^** | -0.96 (-1.44; -0.49)* | -1.45 (-2.05; -0.85)* | -0.26 (-1.03; 0.51) |
| **Waist circumference; cm** | -0.99 (-1.54; -0.45)* | -1.65 (-2.38; -0.93)* | -0.24 (-1.07; 0.59) |
| **Waist to hip ratio** | -0.56 (-1.24; 0.11) | -1.27 (-2.24; -0.30)* | -0.01 (-0.96; 0.94) |
| **Fat mass index; kg/m2** | -1.02 (-1.53; -0.51)* | -1.55 (-2.14; -0.95)* | 0.25 (-0.70; 1.19) |
| **Fat-free mass index; kg/m2** | -0.99 (-1.58; -0.40)* | -1.93 (-2.87; -0.99)* | -0.41 (-1.17; 0.36) |
| **Body cell mass index; kg/m2** | -0.62 (-1.21; -0.03)* | -2.04 (-2.97; -1.11)* | 0.32 (-0.45; 1.10) |
| **Relative fat-free mass; %** | 0.79 (0.14; 1.44)* | 1.69 (0.87; 2.51)* | -0.65 (-1.70; 0.40) |
| **Subcutaneous fat; L** | -0.40 (-1.10; 0.31) | -0.63 (-1.53; 0.28) | 0.06 (-1.14; 1.27) |
| **Visceral fat; L** | -0.51 (-1.24; 0.23) | -2.07 (-3.41; -0.73)* | 0.29 (-0.56; 1.14) |
| **Liver fat; %** | -0.73 (-1.29; -0.17)* | -1.05 (-1.76; -0.33)* | -0.29 (-1.21; 0.63) |

N =1,808 participants (males: 902, females: 906). Results are reported as standardised beta coefficients derived from linear regression models for age, smoking status, years of education, diabetes, fasting glucose, alcohol, physical activity, dietary intake and C-reactive protein. Exposure variables were standardised per standard deviation. EKFC = European Kidney Function Consortia equation; crea+cys = Cystatin C + creatinine, CI = confidence interval, *p<0.05

**Supplementary Material Table 8.** Associations of body composition markers with estimated glomerular filtration rate (eGFR) after additional adjusting for BMI (EKFC_crea+cys_)

|  | **Total**  β (95%-CI) | **Females**  β (95%-CI) | **Males**  β (95%-CI) |
| --- | --- | --- | --- |
| **Body surface area; m^2^** | -0.89 (-1.40; -0.38)* | -1.01 (-1.76; -0.25)* | -0.93 (-1.63; -0.23)* |
| **Waist circumference; cm** | -0.13 (-0.87; 0.61) | -0.59 (-1.58; 0.40) | 0.08 (-1.08; 1.23) |
| **Waist to hip ratio** | 0.33 (-0.11; 0.77) | 0.02 (-0.61; 0.64) | 0.49 (-0.16; 1.14) |
| **Fat mass; kg** | -0.27 (-0.93; 0.39) | -0.56 (-1.55; 0.42) | 0.11 (-0.79; 1.01) |
| **Fat mass index; kg/m2** | 0.04 (-0.83; 0.90) | 0.09 (-1.36; 1.55) | 0.86 (-0.35; 2.08) |
| **Fat-free mass; kg** | -1.12 (-1.76; -0.48)* | -1.91 (-3.12; -0.71)* | -1.39 (-2.21; -0.57)* |
| **Fat-free mass index; kg/m2** | -0.15 (-0.88; 0.57) | -0.30 (-1.55; 0.95) | -0.81 (-1.83; 0.20) |
| **Body cell mass; kg** | -0.82 (-1.44; -0.21)* | -2.39 (-3.51; -1.28)* | -0.38 (-1.18; 0.42) |
| **Body cell mass index; kg/m2** | 0.04 (-0.49; 0.58) | -0.91 (-1.77; -0.05)* | 0.58 (-0.16; 1.31) |
| **Relative fat-free mass; %** | -0.44 (-1.02; 0.13) | -0.57 (-1.43; 0.29) | -0.56 (-1.36; 0.23) |
| **Subcutaneous fat; L** | 0.83 (-0.01; 1.68) | 0.90 (-0.31; 2.11) | 0.86 (-0.32; 2.04) |
| **Visceral fat; L** | -0.18 (-0.86; 0.49) | -1.68 (-3.05; -0.31)* | 0.12 (-0.73; 0.96) |
| **Liver fat; %** | -0.67 (-1.10; -0.24)* | -1.23 (-1.85; -0.60)* | -0.22 (-0.82; 0.37) |

Results are reported as standardised beta coefficients derived from linear regression models adjusted for age, smoking status, years of education, diabetes, fasting glucose, alcohol, physical activity, dietary intake, C-reactive protein and BMI. Exposure variables were standardised per standard deviation. BMI= body mass index; EKFC = European Kidney Function Consortia equation; CKD-EPI equation = Chronic Kidney Disease Epidemiology Collaboration equation, crea = serum creatinine, cys = serum Cystatin C, crea+cys = Cystatin C + creatinine, CI = confidence interval, *p<0.05

**Supplementary Figures: Sex-related standardised beta coefficients for various body composition markers and eGFR in different age groups after additional adjusting for body mass index (BMI)**

**Supplementary Figure 12.** Standardised beta coefficients for waist circumference and eGFR (EKFC_crea+cys_) in different age groups for both sexes. Results are reported as mean with confidence interval for each age group. Linear regression model adjusted for age, smoking status, years of education, diabetes, fasting glucose, alcohol, physical activity, dietary intake, C-reactive protein and BMI, exposure variables standardised per standard deviation. eGFR = estimated glomerular filtration rate; EKFC (CysCrea) = European Kidney Function Consortia equation using Cystatin C + creatinine

**Supplementary Figure 13.** Standardised beta coefficient for waist-to-hip ratio and eGFR (EKFC_crea+cys_) in different age groups. Results are reported as mean with confidence interval for each age group. Linear regression model adjusted for age, smoking status, years of education, diabetes, fasting glucose, alcohol, physical activity, dietary intake, C-reactive protein and BMI, exposure variables standardised per standard deviation. eGFR = estimated glomerular filtration rate; EKFC (CysCrea) = European Kidney Function Consortia equation using Cystatin C + creatinine

**Supplementary Figure 14.** Standardised beta coefficient for body surface area and eGFR (EKFC_crea+cys_) in different age groups. Results are reported as mean with confidence interval for each age group. Linear regression model adjusted for age, smoking status, years of education, diabetes, fasting glucose, alcohol, physical activity, dietary intake, C-reactive protein and BMI, exposure variables Standardised per standard deviation. eGFR = estimated glomerular filtration rate; EKFC (CysCrea) = European Kidney Function Consortia equation using Cystatin C + creatinine

**Supplementary Figure 15.** Standardised beta coefficient for fat mass index and eGFR (EKFC_crea+cys_) in different age groups. Results are reported as mean with confidence interval for each age group. Linear regression model adjusted for age, smoking status, years of education, diabetes, fasting glucose, alcohol, physical activity, dietary intake, C-reactive protein and BMI, exposure variables standardised per standard deviation. eGFR = estimated glomerular filtration rate; EKFC (CysCrea) = European Kidney Function Consortia equation using Cystatin C + creatinine

**Supplementary Figure 16.** Standardised beta coefficient for fat-free mass index and eGFR (EKFC_crea+cys_) in different age groups. Results are reported as mean with confidence interval for each age group. Linear regression model adjusted for age, smoking status, years of education, diabetes, fasting glucose, alcohol, physical activity, dietary intake, C-reactive protein and BMI, exposure variables standardised per standard deviation. eGFR = estimated glomerular filtration rate; EKFC (CysCrea) = European Kidney Function Consortia equation using Cystatin C + creatinine

**Supplementary Figure 17.** Standardised beta coefficient for body cell mass index and eGFR (EKFC_crea+cys_) in different age groups. Results are reported as mean with confidence interval for each age group. Linear regression model adjusted for age, smoking status, years of education, diabetes, fasting glucose, alcohol, physical activity, dietary intake, C-reactive protein and BMI, exposure variables standardised per standard deviation. eGFR = estimated glomerular filtration rate; EKFC (CysCrea) = European Kidney Function Consortia equation using Cystatin C + creatinine

**Supplementary Figure 18.** Standardised beta coefficient for relative fat-free mass and eGFR (EKFC_crea+cys_) in different age groups. Results are reported as mean with confidence interval for each age group. Linear regression model adjusted for age, smoking status, years of education, diabetes, fasting glucose, alcohol, physical activity, dietary intake, C-reactive protein and BMI, exposure variables standardised per standard deviation. eGFR = estimated glomerular filtration rate; EKFC (CysCrea) = European Kidney Function Consortia equation using Cystatin C + creatinine

**Supplementary Figure 19.** Standardised beta coefficient for subcutaneous fat and eGFR (EKFC_crea+cys_) in different age groups. Results are reported as mean with confidence interval for each age group. Linear regression model adjusted for age, smoking status, years of education, diabetes, fasting glucose, alcohol, physical activity, dietary intake, C-reactive protein and BMI, exposure variables standardised per standard deviation. eGFR = estimated glomerular filtration rate; EKFC (CysCrea) = European Kidney Function Consortia equation using Cystatin C + creatinine

**Supplementary Figure 20.** Standardised beta coefficient for visceral fat and eGFR (EKFC_crea+cys_) in different age groups. Results are reported as mean with confidence interval for each age group. Linear regression model adjusted for age, smoking status, years of education, diabetes, fasting glucose, alcohol, physical activity, dietary intake, C-reactive protein and BMI, exposure variables standardised per standard deviation. eGFR = estimated glomerular filtration rate; EKFC (CysCrea) = European Kidney Function Consortia equation using Cystatin C + creatinine

**Supplementary Figure 21.** Standardised beta coefficient for liver fat and eGFR (EKFC_crea+cys_) in different age groups. Results are reported as mean with confidence interval for each age group. Linear regression model adjusted for age, smoking status, years of education, diabetes, fasting glucose, alcohol, physical activity, dietary intake, C-reactive protein and BMI, exposure variables standardised per standard deviation. eGFR = estimated glomerular filtration rate; EKFC (CysCrea) = European Kidney Function Consortia equation using Cystatin C + creatinine

**Supplementary Material Table 9.** Number of participants per CKD stage as per KDIGO guidelines (EKFC_crea+cys_)

|  |  |  | **Albuminuria** | | |  |
| --- | --- | --- | --- | --- | --- | --- |
|  |  |  | **A1** | **A2** | **A3** |  |
|  |  |  | **< 30mg/g** | **30mg/g – 300mg/g** | **> 300mg/g** | **Total** |
| **eGFR in (ml/min/1.73m²)** | **G1** | **> 90** | 2,361 (53.6) | 191 (4.3) | 14 (0.3) | 2,566 (58.2) |
|  | **G2** | **60-89** | 1,361 (30.9) | 239 (5.4) | 31 (0.7) | 1,631 (37.0) |
|  | **G3a** | **45-59** | 109 (2.5) | 37 (0.8) | 13 (0.3) | 159 (3.6) |
|  | **G3b** | **30-44** | 20 (0.5) | 17 (0.4) | 2 (0.1) | 39 (1.0) |
|  | **G4** | **15-29** | 2 (0.05) | 3 (0.1) | 2 (0.1) | 7 (0.3) |
|  | **G5** | **< 15** | 0 (0) | 0 (0) | 1 (0.02) | 1 (0.02) |

Data are expressed as absolute numbers and percentages. CKD= chronic kidney disease; eGFR = estimated glomerular filtration rate; EKFC = European Kidney Function Consortia equation; crea+cys = Cystatin C and creatinine-based equation

**Supplementary Material Table 10.** Correlations between body composition markers

|  | **BMI** | **Waist Circumference** | **Waist-hip ratio** | **Fat mass** | **Fat-free mass** | **Body cell mass** | **Relative fat-free mass** | **Subcutaneous fat** | **Visceral fat** | **Body surface area** |
| --- | --- | --- | --- | --- | --- | --- | --- | --- | --- | --- |
| **BMI (baseline)** | 1.00 |  |  |  |  |  |  |  |  |  |
| **Waist circumference** | 0.83 | 1.00 |  |  |  |  |  |  |  |  |
| **Waist-hip ratio** | 0.45 | 0.79 | 1.00 |  |  |  |  |  |  |  |
| **Fat mass index** | 0.79 | 0.48 | 0.02 | 1.00 |  |  |  |  |  |  |
| **Fat-free mass index** | 0.70 | 0.77 | 0.70 | 0.10 | 1.00 |  |  |  |  |  |
| **Body cell mass index** | 0.55 | 0.63 | 0.64 | -0.03 | 0.92 | 1.00 |  |  |  |  |
| **Relative fat-free mass** | -0.51 | -0.20 | 0.21 | -0.92 | 0.25 | 0.35 | 1.00 |  |  |  |
| **Subcutaneous fat** | 0.80 | 0.58 | 0.13 | 0.90 | 0.25 | 0.11 | -0.78 | 1.00 |  |  |
| **Visceral fat** | 0.69 | 0.85 | 0.73 | 0.35 | 0.69 | 0.55 | -0.12 | 0.44 | 1.00 |  |
| **Body surface area** | 0.58 | 0.75 | 0.57 | 0.17 | 0.74 | 0.70 | 0.09 | 0.33 | 0.63 | 1.00 |

Results are reported as standardised beta coefficients derived from linear regression models. BMI = body mass index.
